# Physicochemical and Theoretical Characterization of a New Small Non-Metal Schiff Base with a Differential Antimicrobial Effect against Gram-Positive Bacteria

**DOI:** 10.3390/ijms23052553

**Published:** 2022-02-25

**Authors:** Manuel Gacitúa, Alexander Carreño, Rosaly Morales-Guevara, Dayán Páez-Hernández, Jorge I. Martínez-Araya, Eyleen Araya, Marcelo Preite, Carolina Otero, María Macarena Rivera-Zaldívar, Andrés Silva, Juan A. Fuentes

**Affiliations:** 1 Facultad de Química y Biología, USACH, Av. L.B. O’Higgins 3363, Santiago 7254758, Chile; manuel.gacitua@usach.cl; 2 Laboratory of Organometallic Synthesis, Center of Applied Nanosciences (CANS), Facultad de Ciencias Exactas, Universidad Andres Bello, República 330, Santiago 8370186, Chile; rmguevara1994@gmail.com (R.M.-G.); dayan.paez@unab.cl (D.P.-H.); 3 Programa de Doctorado en Fisicoquímica Molecular, Facultad de Ciencias Exactas, Universidad Andres Bello, República 275, Santiago 8370146, Chile; 4 Departamento de Ciencias Químicas, Facultad de Ciencias Exactas, Universidad Andres Bello, República 275, Santiago 8370146, Chile; jorge.martinez@unab.cl (J.I.M.-A.); eyleen.araya@unab.cl (E.A.); 5 Departamento de Química Orgánica, Facultad de Química y de Farmacia, Pontificia Universidad Católica de Chile, Santiago 7820436, Chile; mpreite@uc.cl; 6 Escuela de Química y Farmacia, Facultad de Medicina, Universidad Andres Bello, Santiago 8370134, Chile; maria.otero@unab.cl; 7 Laboratorio de Genética y Patogénesis Bacteriana, Facultad de Ciencias de la Vida, Universidad Andres Bello, República 330, Santiago 8370186, Chile; mcr.zaldivar@gmail.com (M.M.R.-Z.); silva.andres.49@gmail.com (A.S.)

**Keywords:** Schiff bases, intramolecular hydrogen bond, HPLC-MS, cyclic voltammetry, DFT, local reactivity analysis, MIC, *Staphylococcus aureus*, *Enterococcus faecalis*, *Bacillus cereus*

## Abstract

Searching for adequate and effective compounds displaying antimicrobial activities, especially against Gram-positive bacteria, is an important research area due to the high hospitalization and mortality rates of these bacterial infections in both the human and veterinary fields. In this work, we explored (*E*)-4-amino-3-((3,5-di-*tert*-butyl-2-hydroxybenzylidene)amino) benzoic acid (SB-1, harboring an intramolecular hydrogen bond) and (*E*)-2-((4-nitrobenzilidene)amino)aniline (SB-2), two Schiff bases derivatives. Results demonstrated that SB-1 showed an antibacterial activity determined by the minimal inhibitory concentration (MIC) against *Staphylococcus aureus*, *Enterococcus faecalis*, and *Bacillus cereus* (Gram-positive bacteria involved in human and animal diseases such as skin infections, pneumonia, diarrheal syndrome, and urinary tract infections, among others), which was similar to that shown by the classical antibiotic chloramphenicol. By contrast, this compound showed no effect against Gram-negative bacteria (*Klebsiella pneumoniae*, *Escherichia coli*, and *Salmonella enterica*). Furthermore, we provide a comprehensive physicochemical and theoretical characterization of SB-1 (as well as several analyses for SB-2), including elemental analysis, ESMS, ^1^H and ^13^C NMR (assigned by 1D and 2D techniques), DEPT, UV-Vis, FTIR, and cyclic voltammetry. We also performed a computational study through the DFT theory level, including geometry optimization, TD-DFT, NBO, and global and local reactivity analyses.

## 1. Introduction

At present, it is universally recognized that the emergence of multiresistant bacteria is an increased public health issue [1]. Multiresistant pathogens have been increasingly reported in human and animal diseases [1,2,3,4]. The Centers of Disease Control and Prevention (CDC) presented a threat report listing multiresistant bacteria that included some important Gram-positive pathogens such as *Clostridioides difficile* (urgent threat), *Enterococci*, *Staphylococcus aureus* (MRSA), *Streptococcus pneumoniae* (serious threats), Group A *Streptococcus*, and Group B *Streptococcus* (concerning threat), among others [1,5]. Gram-positive bacteria present a thick peptidoglycan cell wall protecting the cytoplasmic membrane. In contrast, Gram-negative bacteria exhibit a thin peptidoglycan cell wall and an outer phospholipid membrane harboring proteins and lipopolysaccharides [6]. Thus, the study of novel antimicrobial agents is an active area of research aiming to circumvent the increasing emergence of resistance traits in bacteria showing structural differences. In particular, the use of biocide differential compounds (i.e., which affect only a subset of microorganisms), is attracting interest [7].

Schiff bases are compounds harboring a functional group called azomethine (–C=N–), a linker between structures showing the formula R_1_R_2_–C=N–R_3_, where R1, R2, or R3 could be substituted aryl or alkyl groups, among others [8,9,10]. Schiff bases exhibit a broad range of applications, including anticancer drug candidates [11], where their use in antimicrobial chemotherapy is progressively gaining attention [8,12]. However, the precise mechanism of action supporting the antimicrobial activity is not fully understood, although some works provide evidence dealing with the elucidation of the mode of action (e.g., [13]). Many of the Schiff bases exhibiting antimicrobial activity are coordinated with metals (first transition series and other metals), which are thought to exert this effect [14,15,16,17,18]. Nevertheless, the use of Schiff bases with no metal coordination exhibiting antimicrobial activity has been comparatively less explored [19]. Some advantages of using simpler molecules include their straightforward synthesis, which makes them desirable for pharmaceutical applications [20,21].

**Figure 1:**
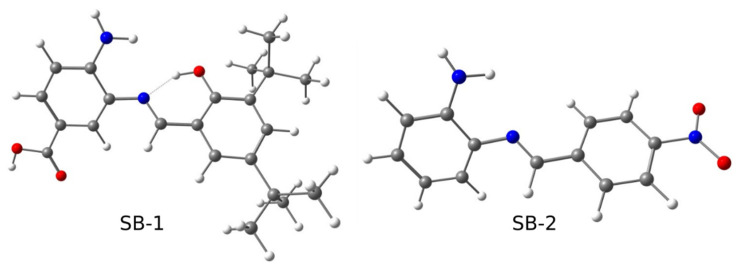
Geometry optimizations of SB-1 and SB-2. Blue: N; red: O; white: H; gray: C.

Our group is particularly interested in non-natural, simple, and small Schiff bases, constituted only by two substituted rings linked by the azomethine group [22]. In particular, we want to understand how to modulate antimicrobial activity by exploring different kinds of substitutions as we previously reported for other Schiff bases [23,24]. In this work, we synthesized and characterized a new Schiff base named SB-1 ((*E*)-4-amino-3-((3,5-di-*tert*-butyl-2-hydroxybenzylidene)amino) benzoic acid], harboring an intramolecular hydrogen bond (IHB), which was compared to SB-2 ((*E*)-2-((4-nitrobenzylidene)amino)aniline] regarding a complete characterization and antimicrobial effects (Figure 1). As stated, SB-1 is a new compound; conversely, SB-2 was already reported as unable to inhibit the bacterial growth of both Gram-positive and Gram-negative bacteria, although its structural characterization was performed only by ^1^H NMR and its electronical characterization was not disclosed [25]. Thus, comparing SB-1 and SB-2 regarding their structural and antimicrobial properties is a good approach to identify potential substitutions participating in biocide activity.

## 2. Results and Discussion

### 2.1. Synthesis and Characterizations

SB-1 and SB-2 (Figure 1) were prepared by direct condensation of a primary amine with their corresponding substituent aldehyde (Schemes S1 and S2) in methanol at room temperature, as described in Methods. SB-1 presented a light brown-yellow color, whereas SB-2 exhibited a dark red color; both compounds were obtained with high yield. SB-1 is an amino-benzoic acid linked by the azomethine group with a phenolic ring, presenting an intramolecular hydrogen bond (IHB) between the OH from the phenolic ring and the N from the Schiff base. On the other hand, SB-2 is a phenylenamine ring linked with a nitrobenzene ring, where the IHB is absent. Both SB-1 and SB-2 presented high solubility in DMSO, lower methanol solubility, and apparent water insolubility at room temperature. Since SB-1 is a new compound, we determined its elemental (C, H, N) microanalysis, which agreed with the formula (C_22_H_28_N_2_O_3_). Furthermore, we performed electrospray mass spectrometry, obtaining the peak at 369.1, in agreement with the proposed molecular formula (Figure S1a). Regarding the melting point, we observed that SB-1 melted at 225–226 °C before decomposition. For SB-2, we also performed elemental analysis (C_13_H_11_N_3_O_2_) and ESMS (Figure S1b), obtaining similar results to those previously reported [25].

In order to analyze the structure of SB-1 and SB-2, we performed NMR experiments in DMSO-d_6_ as solvent (the arbitrary numbering of protons used in the ^1^H NMR studies is shown in (Figure S2).

SB-1 exhibited two narrow signals at 5.78 ppm and 13.36 ppm, assigned to the proton of the amino group (–NH_2_) and the hydroxyl group (–OH), respectively. A less intense and broader signal appeared at 12.24 ppm, assigned to the carboxylic group’s proton (–COOH). All these assignations were confirmed by D_2_O exchange (compare Figures S3 and S4). On the other hand, the azomethine (–CH=N–) proton (H4) appeared as a singlet at 8.93 ppm (Figure S3). The other protons corresponding to the amino-benzoic acid ring appeared at 6.80 ppm (d) (H-2) and 7.68 ppm (s) (H-6). Regarding H1, it appeared as a multiplet overlapped with H-5, at about 7.66–7.52 ppm. Finally, H-6 (found in the phenol ring) appeared at 7.38 ppm (d) (Figure S3). All these assignments were confirmed by HHCOSY (Figure S5). To complement these analyses, the ^13^C NMR spectrum was obtained. We found 14 aromatic carbons between 170 and 113 ppm, whereas *tert*-butyl carbons were found between 36 and 28 ppm. The azomethine carbon was found at 165 ppm (Figure S6). We also performed a DEPT and CHCOSY analysis to corroborate the SB-1 structure (Figures S7 and S8).

Regarding SB-2, the ^1^H NMR was already reported [25], which was corroborated by our data (Figure S9). In this context, we complemented this analysis with a D_2_O exchange, HHCOSY, ^13^C NMR, and DEPT. These analyses were also helpful for validating our procedure of synthesis and in confirming the SB-2 structure. The ^1^H NMR and HHCOSY showed the azomethine proton as a singlet at 8.84 ppm. The four protons in the phenylenamine ring (H1 to H4) appeared at 7.02, 7.24, 6.76, and 6.57 ppm, respectively (Figures S9 and S10). On the other hand, the protons corresponding to the nitrobenzene ring (H6, H6’, H7, and H7’) appeared as two intense signals at 8.32 ppm and 8.27 ppm, indicating that they are homotopic (Figures S9 and S10). The D_2_O exchange corroborated the assignment of the amino group’s protons (–NH_2_) (5.40 ppm; bs; Figure S11). To complement these results and confirm the proposed structure for SB-2, ^13^C NMR, DEPT, and CHCOSY (all in DMSO-d_6_) analyses were performed. In this case, the azomethine carbon was assigned at 154 ppm (see details in Figures S12 and S13a,b).

In order to continue the characterization of both SB-1 and SB-2, the FTIR spectrum (in KBr pellet) was carried out. For SB-1, the FTIR spectrum showed bands that are generally considered symmetric and asymmetric stretching νOH vibrations at 3475.73 cm^−1^ assigned to the IHB (Figure S14). This assignment is consistent with previously reported signals assigned to similar Schiff bases [26]. Two additional bands were observed at 3379.29 and 3059.10 cm^−1^, assigned to the νNH stretching vibrations modes (asymmetric and symmetric) of a primary amino group, in accordance with previously reported results for comparable Schiff bases [26]. We assigned the bands at 2962.66 cm^−1^, 2866.22 cm^−1^, and 2530.61 cm^−1^ as typical νC–H bands. Other absorptions were observed at 1666.50 cm^−1^, 1616.35 cm^−1^, and 1570.06 cm^−1^, assigned as νC=O (carbonyl group), νHC=N (azomethine), and νC=C, respectively. Although the azomethine group is usually assigned to absorptions at around 1640 cm^−1^ [27], the SB-1 azomethine group (1616.35 cm^−1^) can be explained by the presence of the IHB, as described for other similar molecules [26]. SB-2, for its part, showed a characteristic pattern in line with previous studies [25] (Figure S15).

Finally, for SB-1 and SB-2, the UV-Vis spectrum was recorded in methanol at room temperature (Figure S16). In both cases, the spectrum showed two bands; the first centered approximately at 278 nm, was assigned to a mixed of n → π* (–C=N–) and to π → π* transitions; whereas the band approximately at 377 nm was assigned to π → π* transitions, according to previous reports showing other related compounds [28]. In addition, theoretical studies supported all these assignments (see below).

All these analyses supported both the structure and purity of SB-1 and SB-2, indicating that these compounds were suitable for their electrochemical and biological assays.

### 2.2. Electrochemical Characterization

**Figure 2:**
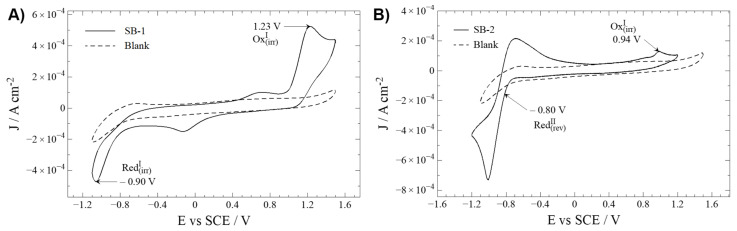
Cyclic voltammetry profiles of Schiff bases (—) and blank (---). Interface: Pt| 10^−3^ mol L^−1^ of compound + 10^−1^ mol L^−1^ of TBAPF_6_ in anhydrous CH_3_CN under an argon atmosphere. Scan rate: 200 mV s^−1^. (**A**) SB-1; (**B**) SB-2.

Electrochemical characterization of novel molecules helps establish a relationship between a molecule structure and physicochemical properties such as oxidation/reduction potentials. Therefore, cyclic voltammetry analyses for SB-1 and SB-2 were performed to elucidate the oxidation and reduction processes. In addition, cyclic voltammograms for these compounds were compared to a blank solution containing TBAPF_6_ in anhydrous CH_3_CN (Figure 2).

Oxidation and reduction processes are studied by direct analysis of the cyclic voltammograms. To distinguish compound-related REDOX processes from secondary reactions with the solvent (e.g., acetonitrile), a working window study is mandatory (Figures S17 and S18) [22]. Similar profiles have been described for similar Schiff bases with an IHB [22,26].

Regarding oxidation, SB-1 and SB-2 display an irreversible oxidation at 1.23 and 0.94 V, respectively. The nature of this irreversible oxidation is typically ascribed to oxidation, taking place somewhere at the aminobenzoic ring, most likely at the –NH_2_ group [24]. The considerable potential difference is probably due to the electron withdrawal influence of the carboxylic acid group on SB-1, which increases the energy (potential) required to oxidize the amine group.

Concerning reduction processes, SB-1 and SB-2 showed peaks at −0.90 (irreversible) and −0.80 V (quasi-reversible). Typically, the reduction processes in this kind of compound are ascribed to an intramolecular reductive coupling of the azomethine group, which involves a self-protonation reaction, as described for other related Schiff bases. This phenomenon can explain the results for SB-1 [22,23,29]. Without dismissing the possibility that the intramolecular reductive coupling of the azomethine group could occur at SB-2, it should be minded that SB-2 possesses a –NO_2_ moiety. Organic nitro-aromatic compounds are known for their intense reversible reduction [30,31], the most likely process observed on this molecule.

**Figure 3:**
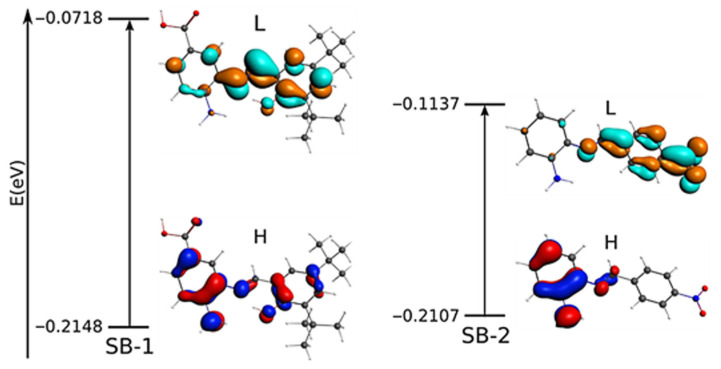
Qualitative molecular orbital diagram for SB-1 and SB-2 with the most important electronic transitions. Occupied orbitals: red and blue; unoccupied orbitals: orange and cyan. L: LUMO; H: HOMO.

To complement these assignations, molecular models and the energy of the frontier orbitals HOMO-LUMO is discussed below. For SB-1, the HOMO distribution analysis, which defines the energy of the highest occupied molecular orbital, mainly comprises the amino in the aminobenzoic ring and the hydroxyl in the phenolic ring (for a qualitative molecular orbital diagram, see Figure 3). On the other hand, in SB-2, the HOMO is mainly located at the phenylenamine ring and, to a lesser extent, the azomethine group, supporting the participation of these groups in oxidation processes. In addition, calculations showed that, for SB-1, the LUMO (lowest unoccupied molecular orbital) is mainly distributed around the azomethine group, which is the region where the electron might be withdrawn during the reduction processes. On the other hand, for SB-2, the LUMO is intensively located over the nitrobenzene ring, which supports the idea that the quasi-reversible reduction of SB-2 takes place at the –NO_2_ moiety. Therefore, the calculation results fairly complement the electrochemical REDOX processes designations.

### 2.3. Geometry Optimizations, TD-DFT, and NBO Studies

In order to complement the experimental data, we performed a geometry optimization. The selected geometry parameter data of SB-1 and SB-2 are listed in Tables S1 and S2, and the optimized geometries are shown in Figures S19 and S20. The optimized structures and geometry parameters agree with available experimental data from a similar Schiff base [29,32]. The azomethine group showed a computed distance and angle degree of 1.286 Å and 121.75° for SB-1; 1.273 Å and 121.88° for SB-2, respectively, in accordance with other Schiff bases [32]. On the other hand, the asymmetric and symmetric vibration mode of –NH_2_ computed appeared at 3732 cm^−1^ and 3626 cm^−1^ for SB-1, and 3696 cm^−1^ and 3582 cm^−1^ for SB-2. The νOH appeared at 3787 cm^−1^ and 3187 cm^−1^ for the –COOH group and IHB observed in SB-1, respectively. For their part, calculations of frequencies showed that the azomethine appeared at 1680 cm^−1^ and 1696 cm^−1^ for SB-1 and SB-2, respectively (Table S3, Figures S21 and S22), corroborating the experimental FTIR assignment (compare with Figures S14 and S15). The calculated energy of the frontier orbital HOMO-LUMO (Figure 3) is reported in eV (E _HOMO−LUMO_ = 0.143 eV for SB-1, and 0.097 eV for SB-2).

To further elucidate the UV-Vis transitions, we conducted time-dependent density functional theory (TD-DFT) calculations for SB-1 and SB-2 in methanol as solvent (Figure S23). The most important absorption bands and other parameters, such as the oscillator strengths and the corresponding transition, are shown in Table S4. The calculated results are in good agreement with experimental data.

To study the IHB of SB-1 in more detail, we evaluated the second-order interaction energy through Natural Bond Orbitals (NBOs) calculations [33]. The interaction energy is related to stabilizing donor-acceptor interactions due to electron delocalization concerning the zeroth-order natural Lewis structure [34]. In addition, NBO analysis provides an efficient method for studying the participation of the IHB in the stability of N-harboring compounds [35]. Thus, from the NBO analysis, the IHB results in stabilization at 26.9 kcal/mol (between the nitrogen lone pairs and an anti-bonding orbital in the –OH group). On the other hand, the oxygen lone pairs can delocalize to the aromatic ring, resulting in stabilization at 39.4 kcal/mol. By contrast, delocalization from the azomethine group to the benzoic acid ring and vice versa contributes to stabilization at 11.1 and 10.9 kcal/mol, respectively. The delocalization from the phenolic ring to the azomethine contributes to stabilizing at 25.2 kcal/mol. These results show that the delocalization among the azomethine group and both aromatic rings provide stability to SB-1. Other interactions involving the –NH_2_ and –COOH substituents are shown in Table S5. For the numbering of atoms for NBO, see Figure S24.

### 2.4. Analysis of Global Reactivity

The chemical reactivity and site selectivity were studied using several reactivity descriptors (more details concerning these descriptors are included in the Supplementary Material). According to Table 1, chemical potential (m) indicates that SB-1 presents a higher escaping tendency of electrons from equilibrium than SB-2. Molecular hardness (h) indicates that SB-1 offers a higher resistance to charge transfer than SB-2; the same is concluded through global softness (S), which reveals that SB-2 presents a more effortless charge transfer than SB-1. These two descriptors are not very clear by themselves; thus, it is necessary to resort to electrophilicity (w), indicating that the energy stabilization due to a maximum electron flow from a donor environment favors SB-2, meaning that this compound would be favored energetically from a nucleophilic attack rather than SB-1. Electron-donating power (*w*^−^) reveals that SB-1 is more likely to donate charge than SB-2. On the contrary, electron-accepting power (*w*^+^) reveals that SB-2 tends to accept a charge more favorably than SB-1. Summing up, these two global descriptors joined together in net electrophilicity indicate that SB-1 is a better electron-donating species than SB-2. Conversely, SB-2 acts better as an electron acceptor than SB-1. For more details regarding these descriptors, please see the Supplementary Material and Figures S25 and S26.

### 2.5. Analysis of Local Reactivity

**Figure 4:**
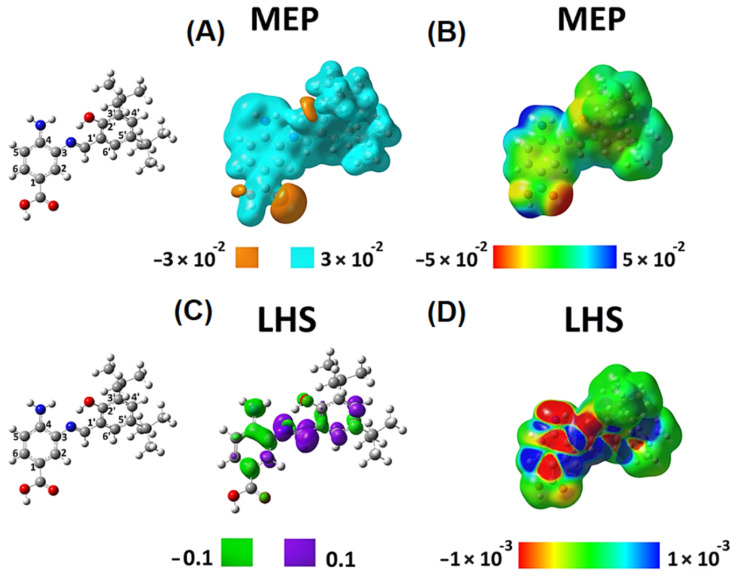
Local reactivity descriptors of SB-1. Molecular electrostatic potential (MEP) and local hyper softness (LHS) values as 3D pictures. Isovalues of MEP are displayed at ±0.03 $hartree\cdot e^{-1\;}$ (or ±0.03 a.u.) (**A**), and isovalues of LHS are displayed at ±0.1 $e^{3\;}\cdot hartree^{-2}\cdot bohr^{-3}$(or ±0.1 a.u.) for LHS (**C**). Values of MEP and LHS projected onto at an electron density isosurface of 0.001 $e\cdot bohr^{-3\;}$ ranging from −0.05 to 0.05 $hartree\cdot e^{-1\;}$ (**B**) and from −0.001 to 0.001 $e^{3\;}\cdot hartree^{-2}\cdot bohr^{-3}$ (**D**) are depicted, respectively. The corresponding atom numbering is shown on the left.

**Figure 5:**
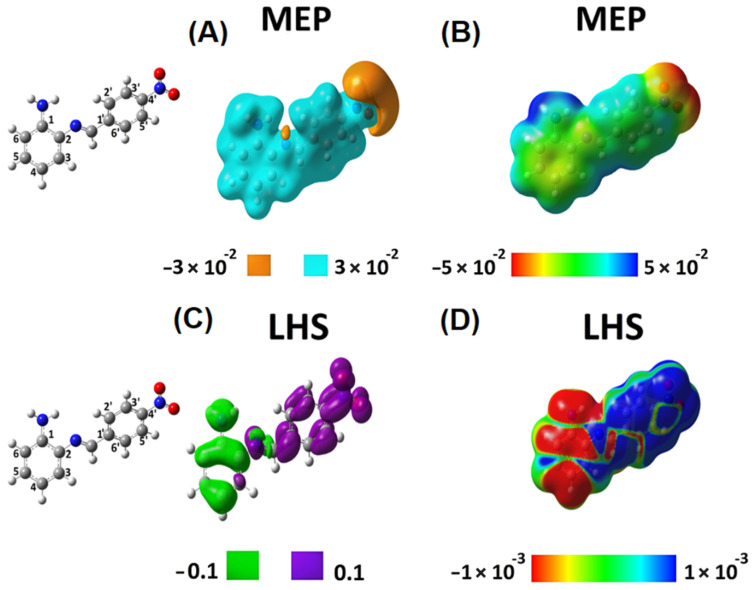
Local reactivity descriptors of SB-2. Molecular electrostatic potential (MEP) and local hyper softness (LHS) as 3D pictures. Isovalues of MEP are displayed at ±0.03 $hartree\cdot e^{-1\;}$ (or 0.03 a.u.) (**A**), and isovalues of LHS are displayed at ±0.1 $e^{3\;}\cdot hartree^{-2}\cdot bohr^{-3}$ (or 0.1 a.u.) for LHS (**C**). Values of MEP and LHS projected onto at an electron density isosurface of 0.001 $e\cdot bohr^{-3\;}$ ranging from −0.05 to 0.05 $hartree\cdot e^{-1\;}$(**B**) and from −0.001 to 0.001 $e^{3\;}\cdot hartree^{-2}\cdot bohr^{-3}$ (**D**), respectively. The corresponding atom numbering is shown on the left.

Density functional theory (DFT) has successfully explained the theoretical background of molecular properties and chemical concepts [36,37]. In this context, we determined descriptors to analyze chemical reactivity and site selectivity of SB-1 and SB-2. To this aim, we obtained the molecular electrostatic potential (MEP) and local hyper softness (LHS) (Figure 4 and Figure 5). Integrated values of LHS are given in $e^{3\;}\cdot hartree^{-2}$. Please see the Supplementary Material (Tables S6–S8) to check the values of condensed LHS atom by atom.

These local reactivity descriptors (MEP and LHS) were projected onto an electron density isosurface of 0.001 $e\cdot bohr^{-3}$ (or 0.001 a.u.) as previously proposed [38], to produce maps that allow for analyzing of how the values of these descriptors evolve along with the entire molecular structure (Figure 4B,D and Figure 5B,D). For SB-1, Figure 4A shows a predominance of electrons on the hydroxyl groups located at the carboxylic function (amino-benzoic acid ring) and the hydroxyl groups of the phenolic ring. The MEP map in Figure 4B confirms the acidic behavior of the hydrogen of the carboxylic function and the subtle acidic behavior of the hydrogens of the amino group. According to this qualitative information, interactions with a cationic species should occur on the hydroxylic groups’ oxygens. By contrast, a species showing an anionic character should interact with the amino group’s hydrogens and more favorably with the carboxylic function’s hydrogens.

The global softness, S, is 7.123 $e^{2}\cdot hartree^{-1}$. According to the LHS descriptor, any possible electrophilic attack will occur on the amino-benzoic acid ring, preferably on the atoms numbered 1, 3, and 4, and the nitrogen of the amino group (Figure 4C). Furthermore, the amino-benzoic ring is the most susceptible region of this molecule to undergo electrophilic attacks. The atoms 1, 3, 4, and 5, along with the nitrogen atom of the amino group, present a total condensed local hyper-softness of -23.79 $e^{3\;}\cdot hartree^{-2}$, while atoms 2 and 6 quantify an amount of 3.17 $e^{3\;}\cdot hartree^{-2}$. These results indicate that this ring is prone to undergo an electrophilic instead of a nucleophilic attack Figure 4D).

On the other hand, the nitrogen atom in the azomethine group and its neighbor carbon sp^2^ atom are prone to nucleophilic attacks. This characteristic behavior is similar to atoms 4′ and 6′ reactivity on the phenolic ring (Figure 4C). On the phenolic ring, the atoms 2′, 3′, 4′, and 6′ give a total condensed local-hyper softness value of 9.79 $e^{3\;}\cdot hartree^{-2}$ and 1′ and 5′ present a total condensed local-hyper softness value of −3.92 $e^{3\;}\cdot hartree^{-2}$. The nitrogen and carbon atoms of the azomethine group present a condensed local-hyper softness value of 18.38 $e^{3\;}\cdot hartree^{-2}$ (Figure 4D). Hence, the amino-benzoic ring is the most reactive molecular fragment of SB-1, and electrophilic attacks should predominate.

The azomethine is the second most reactive site of SB-1, where nucleophilic attacks would be oriented to occur. The *tert-*butyl groups in the phenolic ring show neither electrostatic nor covalent possible interactions, indicating that they could only exert a steric effect, protecting the azomethine group from nucleophilic attacks, facilitating any possible electrophilic attack on the amino-benzoic acid ring.

Concerning SB-2, MEP analysis confirmed that electrons predominate on the nitro group (Figure 5A) and that the hydrogen atoms in the amino group present an acidic behavior (Figure 5B) (as well as in SB-1, Figure 4B). Furthermore, in SB-2, electrophilic attacks will only be oriented towards the phenylenamine ring. Thus, unlike SB-1, SB-2 exhibits a well-separated local reactivity regarding targets for nucleophilic and electrophilic attacks (Figure 5C,D).

For SB-2, the global softness is 9.753 $e^{2}\cdot hartree^{-1}$. The LHS map shows that atoms 1, 2, 4, 5, 6, and nitrogen of the amino group are the most susceptible to electrophilic attacks. In contrast, atoms 1′, 2′, 3′, 4′, 5′, 6′ and the nitro group are susceptible to nucleophilic attacks (Figure 5C,D). After adding the negative integrated local hyper-softness values on the phenylenamine ring (atoms, 1, 2, 4, 5, 6, NH_2_), the resulting value is −75.17 $e^{3\;}\cdot hartree^{-2}$. Conversely, after adding the integrated values of local hyper-softness of the nitrobenzene ring (atoms 1′, 2′, 3′, 4′, 5′, 6′, nitro), we obtained 66.57 $e^{3\;}\cdot hartree^{-2}$. These results show that local reactivities that SB-2 are more favorable than those exhibited by SB-1. Therefore, SB-1 and SB-2 exhibit different local reactivity properties, suggesting that these compounds present, in turn, different biological activities. To end this theoretical analysis, we confirmed the presence of an IHB in SB-1 through Wiberg and Mayer bond order analyses. The same analysis confirmed no IHB in SB-2 (Figures S27 and S28).

### 2.6. Analysis of Antimicrobial Activity

Previously, it has been described that SB-2 exerted no antimicrobial effect against both Gram-positive and Gram-negative bacteria, including *Staphylococcus aureus*, *Escherichia coli,* and *Klebsiella* sp. [25]. This information was corroborated by determining the minimal inhibitory concentration (MIC) of Gram-positive and Gram-negative bacteria (Table 2; see a resistance/sensitivity profile of tested bacteria in Tables S9 and S10). However, when SB-1 was tested, we found that this compound exerted a potent antimicrobial activity (comparable to that shown by the antibiotic chloramphenicol, which affects protein synthesis [39]) only against Gram-positive pathogens (Table 2). Gram-positive and Gram-negative differ essentially in the configuration of their cell envelopes. Gram-positive bacteria present a cytoplasmic membrane surrounded by a thick cell wall, whereas Gram-negative bacteria have an inner membrane, a thin cell wall, and a second outer membrane over the wall. In both cases, the cell wall is constituted of peptidoglycan [40,41]. The intrinsic resistance of Gram-negative bacteria could explain this differential antimicrobial activity of SB-1 over Gram-positive bacteria due to the exclusion of the compound by the presence of the outer membrane. In this sense, Gram-negative bacteria exhibit intrinsic resistance to vancomycin because of the limit of diffusible molecules through the outer membrane [42]. Previously, it has been reported that *Salmonella* Typhi Δ*ompA* mutant (which lacks the porin OmpA) and *Salmonella* Typhi Δ*yibP* (Δ*envC*) mutant (which affect the peptidoglycan hydrolysis) present an increased envelop permeability, which leads to a susceptibility to vancomycin [43]. In this context, we also tested the antimicrobial activity of SB-1 against the *Salmonella* Typhi Δ*ompA* and Δ*yibP* (Δ*envC*) mutants; nevertheless, we found no effect (data not shown). However, it is necessary to underline that, despite mutations affecting permeability, these mutants still present the outer membrane. That structure could hamper the uptake of the SB-1, impairing the contact with its target. Thereby, we postulate that the presence of the outer membrane, present only in Gram-negative bacteria, could explain why SB-1 cannot efficiently kill this kind of bacteria. Thus, more experimentation is needed to unequivocally determine the molecular bases of the differential antimicrobial activity of SB-1 against Gram-positive bacteria.

As described above, SB-1 is constituted by two rings (amino-benzoic acid ring and the phenolic ring) connected by the azomethine. To assess whether the antimicrobial effect can be attributed to one of these moieties, we tested the precursors for SB-1 synthesis, i.e., A: 3,4-diaminobenzoic acid, and B: 3,5-di-*tert*-butyl-2-hydroxybenzaldehyde (Scheme S1) against two Gram-positive and one Gram-negative bacteria. Interestingly, we found that none of these precursors presented antimicrobial activity, even against Gram-positive bacteria that showed to be highly susceptible to SB-1. However, since SB-2 also presents an azomethine group, the antimicrobial effect is not plausibly attributed to this part of the compound. Thus, all these results support that the whole structure of SB-1, and not the rings separately, are responsible for the antimicrobial activity, reinforcing the importance of the relationship between structure and activity in this kind of compound [23,24]. In this context, the IHB, which contributes to the SB-1 structure, might influence this compound’s antimicrobial activity.

Thus, SB-1 showed a potent antimicrobial activity only against Gram-positive bacteria (which exhibit resistance to other antibiotics, e.g., penicillin or tetracycline (Table S9), including *Staphylococcus aureus*, *Bacillus cereus*, and *Enterococcus faecalis* (Table 2). *Staphylococcus aureus* is a non-sporulated pathogen frequently isolated in hospital facilities, which is the etiologic agent of skin, soft tissue, and bone infections. This pathogen is also involved in endocarditis, bacteremia, and toxic shock syndrome, all deadly conditions [44]. *Bacillus cereus* is a spore-forming ubiquitous pathogen mainly involved in food poisoning [45]. *Enterococcus faecalis* is a non-sporulated Gram-positive pathogen that colonizes the gastrointestinal tract of humans and animals. This pathogen is considered an important agent for hospital-acquired infections, mainly affecting susceptible patients (i.e., immunocompromised patients) [46]. In addition, *Staphylococcus aureus* and *Enterococcus faecalis* are considered a serious threat by the Centers of Disease Control and Prevention regarding the emergence of multiresistant strains [1,5]. Thus, SB-1 could be considered a promising compound to be explored as a potential antimicrobial agent against these pathogens.

## 3. Materials and Methods

### 3.1. Materials and Instruments

The reagents used for the synthesis were commercially available by Aldrich, and they were used without further purification. FTIR spectra were obtained on a Tracer-100 FT-IR spectrophotometer (Shimadzu, Kyoto, Japan) with a KBr pellet. ^1^H NMR, HHCOSY, ^13^C NMR, CHCOSY, and DEPT 45 for SB-1 and/or SB-2 were recorded on a Bruker AVANCE 400 spectrometer (Bruker, Billerica, MA, USA) (400 MHz for ^1^H and 100 MHz for ^13^C) at 25 °C and are reported in ppm downfield from TMS. Unless otherwise stated, samples were dissolved in deuterated DMSO containing tetramethyl silane as an internal reference. Melting points were determined on a Stuart Scientific melting point apparatus SMP3 (Stuart, Staffordshire, UK) in open capillary tubes and were uncorrected. Elemental analyses (CHNS) were performed in a Thermo Flash 2000 Series (Thermo Scientific, Waltham, MA, USA), with a thermal conductivity detector (TCD). In addition, ESMS (electrospray mass spectrometry) of SB-1 was performed with a MS 2020 instrument (Shimadzu, Kyoto, Japan). For the electrochemical experiments, a working solution contained 10^−3^ mol L^−1^ of the respective compound (SB-1 and SB-2) with 10^−1^ mol L^−1^ tetrabutylammonium hexafluorophosphate (TBAPF_6_, supporting electrolyte) in anhydrous CH_3_CN. Before each experiment, the working solution was purged with high purity argon, and an argon atmosphere was maintained during the experiment, as previously reported [24,47]. A polycrystalline non-annealed platinum disc (2 mm diameter) was used as the working electrode. A platinum gauze of a large geometrical area, separated from the cell’s main compartment by a fine sintered glass, was used as the counter electrode. All potentials quoted in this paper refer to an Ag/AgCl electrode in tetramethylammonium chloride to match the potential of a saturated calomel electrode (SCE) at room temperature. All electrochemical experiments were performed at room temperature on a CHI900B bipotentiostat (CH instruments, Austin, TX, USA) interfaced to a PC running the CHI 9.12 software (CH instruments, Austin, TX, USA) that allowed experimental control and data acquisition [48,49].

### 3.2. Procedure for Preparing SB-1 and SB-2

SB-1 and SB-2 were prepared by direct interaction between the corresponding amino and aldehyde compounds at room temperature and stirring. As a result, the synthesis yield was more than 75% in both cases.

#### 3.2.1. Synthesis of (*E*)-4-Amino-3-((3,5-di-*tert*-butyl-2-hydroxybenzylidene)amino) Benzoic Acid (SB-1)

The synthesis was performed by condensation between 3,4-diaminobenzoic acid (precursor A) and 3,5-di-*tert*-bytyl-2-hydroxy-benzaldehyde (precursor B) (1:1) in 20 mL of methanol as previously described for similar synthesis [22,29]. The reaction was stirred for 24 h at room temperature, without the need of heating or an inert atmosphere. The precipitate was filtered, washed with ethanol and diethyl ether (50/50 *v/v*), and vacuum dried. A light brown-yellow amorphous compound was obtained (yield: 79%. m.p. = 225–226 °C, dec.). FTIR (KBr, cm^−1^): 3475 (νOH), 3379 and 3059 (νNH_2_), 2962 (νC=C), 1666 (νC=O), 1616 (νN=C), 1570 (νC=C). ^1^H NMR δ = 5.78 (bs; 2H; NH_2_), 6.80 (d; J = 8.4; 1 H, H2), 7.38 (s; 1H; H6), 7.66–7.52 (m; 2H; H1 and H5), 7.68 (s; 1H; H3), 8.93 (s; 1H; H4), 12.24 (s, 1H; -COOH), 13.36 (s, 1H; -OH).^13^C NMR δ = 167.72, 164.71, 157.64, 147.32, 140.92, 136.25, 133.61, 129.93, 128.56, 127.58, 120.59, 119.23, 118.85, 114.87, 35.28, 34.29, 31.68, and 29.93; DEPT: δ = 164.72, 129.82, 128.30, 127.55, 120.67, 114.65, 31.60, and 29.80. UV/Vis: (methanol, room temperature) λ nm (ε mol^−1^ dm^3^ cm^−1^): 278 (93.643 × 10^4^), 377 (12.695 × 10^3^). Anal. Calcd. (%) for C_22_H_28_N_2_O_3_: C, 71.71; H, 7.66; N, 7.60; C/N = 9.43. Found (%): C, 70.46; H, 7.65; N, 7.55, C/N = 9.33. EI-MS (ESI+): m/z calculated for C_22_H_28_N_2_O_3_ = 368.4, found (M+H)^1+^ = 369.1.

#### 3.2.2. Synthesis of (*E*)-2-((4-Nitrobenzylidene)amino)aniline (SB-2)

The synthesis was performed as previously reported [24], with slight modification. Briefly, a condensation between 4-nitrobenzaldehyde and 1,2-phenylenediamine (1:1) was carried out as described 2.2.1. A dark red amorphous compound was obtained (yield: 76%). FTIR (KBr, cm^−1^): 3464 and 3367 (νNH_2_), 1608 (νN=C), 1597 (νC=C), 1454 (νC=C), 1342 (νC-N), 1315 (νNO_2_). ^1^H NMR: δ = 5.40 (bs; 2H; -NH_2_), 6.67–6.43 (m; 1 H), 6.75 (d; 1H), 7.02 (t; 1H), 7.24 (d; 1H), 8.30 (dd; 4H), 8.84 (s, 1H). ^13^C NMR: δ = 153.98, 148.83, 145.41, 142.93, 134.47, 129.88, 129.28, 124.34, 117.51, 116.45, and 115.59; DEPT: δ = 153.96, 129.67, 129.28, 124.33, 117.51, 116.45, and 115.52. UV/Vis: (methanol, room temperature) λ nm (ε mol^−1^ dm^3^ cm^−1^): 281 (31.120 × 10^3^), 416 (11.447 × 10^3^). Anal. Calcd. (%) for C_13_H_11_N_3_O_2_: C, 64.66; H, 4.55; N, 17.40; C/N = 3.71. Found (%): C, 62.12; H, 4.22; N, 16.56, C/N = 3.75. EI-MS (ESI+): m/z calculated for C_13_H_11_N_3_O_2_ = 241.2, found (M-H) = 240.0; (M+K^+^)^1+^ = 281.2.

### 3.3. Computational Details

The analyses of local reactivity, geometrical optimizations, frequency, and TD-DFT (in methanol) were performed by the B3LYP functional with no symmetry restrictions and using the 6-311G(d,p) basis set [50,51,52,53]. In addition, frequency calculations were performed to identify the stationary points as minima, where we did not find imaginary frequencies. All calculations were carried out using the Gaussian 09 software package (Gaussian, Wallingford, CT, USA).

We computed 3D pictures of molecular electrostatic potentials (MEP) [54,55,56] and local hyper-softness (LHS) at the B3LYP/6-311++G(d,p) level of theory [54,55,56,57]; however, MEP is routinely computed by using Gaussian 09. To calculate LHS, Cubegen and Cubman programs (Gaussian, Wallingford, CT, USA) were used. Nevertheless, the integrated values of LHS were obtained using the AOMix 6.94 software (SG-Chem, Ottawa, ON, Canada) based on single-point calculations obtained at the B3LYP/6-311G(d,p) level of theory, thus meaning that they do not include diffuse functions as a demanded constrain by AOMix. After applying this constrain, the integration of Fukui functions was performed as follows (Equations (1) and (2)):

(1) $$f_{k}^{+}=\underset{a\;\in \;k}{\sum }\left[C_{a\;i}^{2}+C_{a\;i}\underset{b\;\notin \;k\;}{\sum }C_{b\;i}S_{ab}\right]$$

(2) $$f_{k}^{-}=\underset{a\;\in \;k}{\sum }\left[C_{a\;j}^{2}+C_{a\;j}\underset{b\;\notin \;k\;}{\sum }C_{b\;j}S_{ab}\right]$$

where $C_{ai}^{2}$ are the squared coefficients defining the linear combination of atomic orbitals that produces the *i*th molecular occupied orbital and $C_{aj}^{2}$ are the squared coefficients defining the linear combination of atomic orbitals that produces the *j*th molecular virtual orbital. While $S_{ab}$ is an overlap integral between two atomic functions, it has nothing to do with the global softness S, and when i=LUMO and j=HOMO that leads to (Equations (3) and (4)):

(3) $$f_{k}^{+}=\underset{a\;\in \;k}{\sum }\left[C_{a\;LUMO}^{2}+C_{a\;LUMO}\underset{b\;\notin \;k\;}{\sum }C_{b\;LUMO}S_{ab}\right]$$

(4) $$f_{k}^{-}=\underset{a\;\in \;k}{\sum }\left[C_{a\;HOMO}^{2}+C_{a\;HOMO}\underset{b\;\notin \;k\;}{\sum }C_{b\;HOMO}S_{ab}\right]$$

The given values of $f_{k}^{+}$ and $f_{k}^{-}$ correspond to the Fukui indexes ranging from 0 to 1 each. The dual descriptor index is (Equation (5)):

(5) $$f_{k}^{\left(2\right)}=\underset{a\;\in \;k}{\sum }\left[C_{a\;LUMO}^{2}+C_{a\;LUMO}\underset{b\;\notin \;k\;}{\sum }C_{b\;LUMO}S_{ab}\right]-\underset{a\;\in \;k}{\sum }\left[C_{a\;HOMO}^{2}+C_{a\;HOMO}\underset{b\;\notin \;k\;}{\sum }C_{b\;HOMO}S_{ab}\right]$$

It ranges from −1 to 1, and after integrating all over the atomic domain $\Omega_{k}$, the resulting value will be defined in the interval (−1–1). A positive value within the (0–1) interval indicates that the molecular region is prone to undergo nucleophilic attacks; on the contrary, a negative value within the (−1–0) interval reveals that the zone is prone to undergo electrophilic attacks. The condensed LHS on the *k*th atom is obtained through the following operational formula (Equation (6)):

(6) $$s_{k}^{\left(2\right)}\approx S^{2}f_{k}^{\left(2\right)}=S^{2}\underset{a\;\in \;k}{\sum }\left[C_{a\;LUMO}^{2}-C_{a\;HOMO}^{2}+C_{a\;LUMO}\underset{b\;\notin \;k\;}{\sum }C_{b\;LUMO}S_{ab}-C_{a\;HOMO}\underset{b\;\notin \;k\;}{\sum }C_{b\;HOMO}S_{ab}\right]$$

Then, $s_{k}^{\left(2\right)}$ ranges from negative to positive values under the following restriction (Equation (7)):

(7) $$\overset{M}{\underset{k=1}{\sum }}s_{k}^{\left(2\right)}=0$$

The latter is a consequence of the “normalization” to zero of LHS when integrated on all space and not only on an atomic domain $\Omega_{k}$, $\int s^{\left(2\right)}\left(r\right)\;dr=0$; but this is a consequence of its approximated operational formula $S^{2}f_{k}^{\left(2\right)}$. The complete operational formula does not obey the restriction mentioned above.

More details concerning these descriptors are exposed in the Supplementary Material.

### 3.4. Antimicrobial Activity

SB-1 and SB-2, as well as the precursors for SB-1 (A: 3,4-diaminobenzoic acid, and B: 3,5-di-*tert*-butyl-2-hydroxybenzaldehyde) were evaluated for their in-vitro growth inhibitory activity against the Gram-positive, clinical isolates *Staphylococcus aureus*, *Bacillus cereus*, *Enterococcus faecalis*, from the Hospital Clínico de la Universidad de Chile (Santiago de Chile, Chile) (Table S9). In addition, we also tested the Gram-negative pathogens *Salmonella enterica* subsp. *enterica* serovar Typhimurium (ATCC14028s) [58,59], *Salmonella enterica* subsp. *enterica* serovar Typhi STH2370 [43,60,61] and clinical isolates from the Hospital Clínico de la Universidad de Chile (*Escherichia coli* and *Klebsiella pneumoniae*) (Table S2). Minimum inhibitory concentration (MIC) was obtained by broth dilution essentially as described [62]. MIC is defined as the lowest concentration of the tested compounds at which no growth of the strain was observed after the incubation [62]. Microorganisms were previously cultured in Luria-Bertani broth (LB, Bacto peptone, 10 g/L; Bacto yeast extract, 5 g/L; NaCl, 5 g/L) at 37 °C with shaking to OD_600_ = 1.4 (stationary phase). Further dilutions of microorganisms (0.5 McFarland) were performed with Bacto Tryptic Soy broth (pancreatic digest casein 17.0 g/L, papaic digest of soybean 3.0 g/L, dextrose 2.5 g/L, sodium chloride 5.0 g/L, dipotassium phosphate 2.5 g/L). Stock solutions of the tested compounds were prepared in dimethyl sulfoxide (DMSO) or ethanol 95% for chloramphenicol (Cam, antibiotic used as positive control). The inoculated wells were incubated at 37 °C for 24 h. As control, DMSO alone was used. Only when the tested compounds’ inhibition effect was higher than the effect of DMSO alone was the compound considered to exhibit detectable antimicrobial activity. The MIC values of the tested compounds were obtained as µM. All the experiments were performed in biological and technical triplicate in each assay. For details of dilutions used for MIC calculation, see Table S11.

## 4. Conclusions

In this work, we synthesized and characterized SB-1, and compared it with SB-2. We found that only SB-1 exhibited an intramolecular hydrogen bond (IHB), corroborated by ^1^H NMR, D_2_O exchange, and FTIR. Furthermore, we found that SB-1 exerted a potent antimicrobial activity only against Gram-positive pathogens. Nevertheless, this antimicrobial activity cannot be attributed to their precursor rings separately. Thus, this result is promising regarding the synthesis of new potential antimicrobial agents against bacteria considered to be a serious threat due to the emergence of multiresistant strains.

## Figures and Tables

**Table 1:** Some global reactivity descriptors and SB-1, SB-2, and precursors for SB-1 were obtained from the B3LYP/6-311++G(d,p) level of theory.

Global Reactivity Descriptors		FDA		FMOA	
Name	Symbol	SB-1	SB-2	SB-1	SB-2
First Vertical Ionization Potential	*I_1_*	0.2661	0.2763	0.2175	0.2171
Second Vertical Ionization Potential	*I_2_*	0.4115	0.4521	0.2175	0.2171
First Vertical Electron Affinity	*A_1_*	0.0264	0.0614	0.0766	0.1172
Second Vertical Electron Affinity	*A_2_*	−0.0919	−0.0879	0.0766	0.1172
Electronic Chemical Potential	*µ*	−0.1463	−0.1688	−0.1471	−0.1671
Molecular Hardness	*η*	0.2397	0.2149	0.1409	0.1000
Global Softness	*S*	4.1722	4.6528	7.0977	10.0030
Electrophilicity	*ω*	0.0446	0.0663	0.0768	0.1397
Electron donating Power	*ω* ^−^	0.1774	0.2305	0.2359	0.3693
Electron donating Power	*ω* ^+^	0.0311	0.0617	0.0888	0.2021
Net Electrophilicity	Δ*ω*	0.2085	0.2921	0.3247	0.5714

Notice that *I_1_* and *A_1_* in the FMOA column correspond to the frontier molecular orbitals energies under the assumption that Koopmans’ theorem is satisfied.

**Table 2:** Minimal inhibitory concentration (MIC) (µM) of SB-1, SB-2, and precursors for SB-1 (called A and B). Bacteria were cultured for 24 h before MIC determination (*n* = 3).

			Schiff Bases		Precursors of SB-1	
Strains	Gram	Cam ^a^	SB-1	SB-2	A ^b^	B ^c^
*Staphylococcus aureus*	Positive	3.9 ± 3.7	7.8 ± 2.9	NE	NE	NE
*Bacillus cereus*	Positive	7.8 ± 5.8	3.9 ± 3.1	NE	NE	NE
*Enterococcus faecalis*	Positive	3.9 ± 5.9	7.8 ± 5.7	NE	ND ^e^	ND
*Klebsiella pneumoniae*	Negative	7.8 ± 0.1	NE ^d^	NE	ND	ND
*Salmonella Typhimurium*	Negative	7.8 ± 3.7	NE	NE	NE	NE
*Salmonella Typhi*	Negative	7.8 ± 7.8	NE	NE	ND	ND
*Escherichia coli*	Negative	7.8 ± 3.1	NE	NE	ND	ND

^a^ Cam: Chloramphenicol (positive control). ^b^ A: 3,4-diaminobenzoic acid (precursor corresponding to the aminobenzoic acid ring of SB-1). ^c^ B: 3,5-di-*tert*-butyl-2-hydroxybenzaldehyde (precursor corresponding to the phenolic ring of SB-1) ^d^ NE: No effect (indistinguishable from the vehicle alone, i.e., DMSO) ^e^ ND: Nondetermined.

## Supplementary Materials

The following supporting information can be downloaded at: https://www.mdpi.com/article/10.3390/ijms23052553/s1.
