# Supplementary material for: Physicochemical and Theoretical Characterization of a New Small Non-Metal Schiff Base with a Differential Antimicrobial Effect against Gram-Positive Bacteria
Source: Int J Mol Sci. 2022 Feb 25;23(5):2553. doi: 10.3390/ijms23052553 (PMC8910636; doi:10.3390/ijms23052553)
Supplement: Supplementary file 1 [file ijms-23-02553-s001.zip › ijms-1573486-supplementary.pdf]

SUPPLEMENTARY INFORMATION

Physicochemical and Theoretical Characterization of a New Small Non-Metal Schiff Base with a Differential Antimicrobial Effect against Gram-Positive Bacteria

Manuel Gacitúa [1], Alexander Carreño [2,3,*], Rosaly Morales-Guevara [2,3], Dayán Páez-Hernández [2,3], Jorge I. Martínez-Araya [4], Eyleen Araya [4], Marcelo Preite [5], Carolina Otero [6], María Macarena Rivera-Zaldívar [7], Andrés Silva [7] and Juan A. Fuentes [7,*]

[1] Facultad de Química y Biología, USACH, Av. L.B. O'Higgins 3363, Santiago 7254758, Chile

[2] Laboratory of Organometallic Synthesis, Center of Applied Nanosciences (CANS), Facultad de Ciencias Exactas, Universidad Andres Bello, República 330, Santiago 8370186, Chile; rmguevara1994@gmail.com (R.M.-G.); dayan.paez@unab.cl (D.P.-H.)

[3] Programa de Doctorado en Fisicoquímica Molecular, Facultad de Ciencias Exactas, Universidad Andres Bello, República 275, Santiago 8370146, Chile

[4] Departamento de Ciencias Químicas, Facultad de Ciencias Exactas, Universidad Andres Bello, República 275, Santiago 8370146, Chile; jorge.martinez@unab.cl (J.I.M.-A.); eyleen.araya@unab.cl (E.A.)

[5] Departamento de Química Orgánica, Facultad de Química y de Farmacia, Pontificia Universidad Católica de Chile, Santiago 7820436, Chile; mpreite@uc.cl

[6] Escuela de Química y Farmacia, Facultad de Medicina, Universidad Andres Bello, Santiago 8370134; ma-ria.otero@unab.cl

[7] Laboratorio de Genética y Patogénesis Bacteriana, Facultad de Ciencias de la Vida, Universidad Andres Bello, República 330, Santiago 8370186, Chile; mcr.zaldivar@gmail.com (M.M.R.-Z.); silva.andres.49@gmail.com (A.S.)

* Correspondence: alexander.carreno@unab.cl (A.C.); jfuentes@unab.cl (J.A.F.)

Conceptual Density Functional Theory

Global reactivity.

Several reactivity descriptors can be proposed from the Conceptual Density Functional Theory [31, 32]. Those that do not depend upon position vector $\mathbf{r}$ are called global reactivity descriptors. In the first place, we have the electronic chemical potential $\mu = \left(\frac{\partial E}{\partial N}\right)_{\upsilon(\mathbf{r})} = -\chi$ (**Equation S1)** which is expressed in $hartree \cdot e^{-1}$ in agreement with its definition, E is the energy, N is the number of electrons and χ is the electronegativity. It measures the escaping tendency of electrons from equilibrium and whose operational formula based on the finite difference approximation (FDA) is $\mu = -0.5(I_1 + A_1)$ (**Equation S2**) where I_1 is the first vertical ionization potential, and A_1 is the first vertical electron affinity. While the frontier molecular orbital approximation (FMOA) based on the Koopmans' theorem leads to the following operational formula $\mu \approx 0.5(\varepsilon_H + \varepsilon_L)$ (**Equation S3**) where ε_H stands for the HOMO energy; ε_L, for the LUMO energy.

The molecular hardness, $\eta = \left(\frac{\partial^2 E}{\partial N^2}\right)_{\upsilon(\mathbf{r})}$ (**Equation S4**) is expressed in $hartree \cdot e^{-2}$ and it is understood as the resistance to charge transfer; its operational formula based on the FDA is $\eta = I_1 - A_1$ (**Equation S5**) and after applying the Koopmans' theorem, we obtain $\eta \approx \varepsilon_L - \varepsilon_H$ (**Equation S6**). The global softness is formally defined as $S = \eta^{-1} = \left(\frac{\partial^2 E}{\partial N^2}\right)^{-1}_{\upsilon(\mathbf{r})}$(**Equation S7**) and it is expressed in $hartree^{-1} \cdot e^{2}$; it quantifies the ease to charge transfer of a system, its operational formula, based on the FDA, is $S = (I_1 - A_1)^{-1}$(**Equation S8**). In terms of frontier molecular orbitals energies, we get $S \approx (\varepsilon_L - \varepsilon_H)^{-1}$ (**Equation S9**). The molecular hyper hardness, $\gamma = \left(\frac{\partial^3 E}{\partial N^3}\right)_{\upsilon(\mathbf{r})}$ (**Equation S10**) is expressed in $hartree \cdot e^{-3}$ and whose physical meaning and relevance is under study [1]. Even so, its operational formula based on the FDA is given by $\gamma = 0.5(I_1 + A_1 - I_2 - A_2)$ (**Equation S11**) and usually its absolute value is smaller than

absolute values of μ and η. After applying the FMOA, its value is exactly equals to zero: $\gamma = 0.5(\varepsilon_H + \varepsilon_L - \varepsilon_H - \varepsilon_L) = 0$ (**Equation S12**).In order to take into account the electronic chemical potential and the molecular hardness at the same time, the electrophilicity index ω was proposed as $\omega = \frac{\mu^2}{2\eta}$ (**Equation S13**) [2]. It measures the energy change of an electrophile when it becomes saturated with electrons by considering the case when an electrophilic species is immersed in an idealized zero-temperature free-electron sea of zero chemical potential. Its operational formula corresponds to $\omega = \frac{(I_1+A_1)^2}{8(I_1-A_1)}$ (**Equation S14**) which is based on the FDA, after applying the FMOA, it turns into $\omega \approx \frac{(\varepsilon_H+\varepsilon_L)^2}{8(\varepsilon_L-\varepsilon_H)}$ (**Equation S15**). In order to quantify the response to charge donation and charge acceptance, the electron-donating and electron-accepting powers are defined as follows, respectively: $\omega^- = \frac{(\mu^-)^2}{2\eta^-}$ (**Equation S16**) and $\omega^+ = \frac{(\mu^+)^2}{2\eta^+}$ (**Equation S17**). It works as follows: a smaller value of ω^- of a system makes it a better electron donor, whereas a larger ω^+ value corresponds to a better capability of accepting the charge. Operational formulae are defined as follows $\omega^- = \frac{(3I_1+A_1)^2}{16(I_1-A_1)}$ (**Equation S18**) and $\omega^+ = \frac{(I_1+3A_1)^2}{16(I_1-A_1)}$ (**Equation S19**) which are based on the FDA. When the FMOA is applied, these formulae turn into $\omega^- \approx \frac{(3\varepsilon_H+\varepsilon_L)^2}{16(\varepsilon_L-\varepsilon_H)}$ (**Equation S20**) and $\omega^+ \approx \frac{(\varepsilon_H+3\varepsilon_L)^2}{16(\varepsilon_L-\varepsilon_H)}$ (**Equation S21**) [3]. In order to quantify these both capabilities in just one term, the net electrophilicity $\Delta\omega^\pm$ was proposed, and it corresponds to an electron-accepting power relative to electron-donating power; its operational formula is given by: $\Delta\omega^\pm = \omega^+ - (-\omega^-) = \omega^+ + \omega^-$ (**Equation S22**). Operational formulae based on FDA or FMOA, depend on the respective operational formulae of ω^+ and ω^- as defined before [4].

Local reactivity: Local Hyper-softness

From the Conceptual Density Functional Theory [5, 6], a local reactivity descriptor that reveals sites on a molecule that is susceptible to undergo nucleophilic and electrophilic attacks trending to form covalent bonds is the Fukui function, which presents two forms: the nucleophilic Fukui function $f^{+}(\mathbf{r})$ (to quantify nucleophilic attacks) and the electrophilic Fukui function $f^{-}(\mathbf{r})$ (to quantify electrophilic attacks) [7]. They are scalar fields ranging from 0 to 1, and, under the FDA, they are written in terms of electron densities. However, under the FMOA, their operational formulae are expressed in terms of frontier molecular orbitals as follows:

$$f^{+}(\mathbf{r}) = \rho_{N+1}(\mathbf{r}) - \rho_{N}(\mathbf{r}) \approx |\psi_{LUMO}(\mathbf{r})|^2 \quad \textbf{(Equation S23)}$$

$$f^{-}(\mathbf{r}) = \rho_{N}(\mathbf{r}) - \rho_{N-1}(\mathbf{r}) \approx |\psi_{HOMO}(\mathbf{r})|^2 \quad \textbf{(Equation S24)}$$

where $|\psi_{LUMO}(\mathbf{r})|^2$ is the electron density of LUMO and $|\psi_{HOMO}(\mathbf{r})|^2$ is the electron density of HOMO. These parameters can be plotted as 3D isosurfaces revealing electrophilic and nucleophilic regions on a molecule, respectively; its unit is $bohr^{-3}$.

To avoid the use of these two Fukui functions separately, a second-order Fukui function, also called dual descriptor, has been defined by Christophe Morell *et al.* [8, 9]. Its operational formula is presented as follows:

$$f^{(2)}(\mathbf{r}) = \rho_{N+1}(\mathbf{r}) - 2\rho_{N}(\mathbf{r}) + \rho_{N-1}(\mathbf{r}) \approx |\psi_{LUMO}(\mathbf{r})|^2 - |\psi_{HOMO}(\mathbf{r})|^2 \quad \textbf{(Equation S25)}$$

Its unit is $e^{-1}bohr^{-3}$ where e means electron. It ranges from -1 to 1.

Since chemists are interested in numerical values associated with one or more atoms on a molecule instead of numerical values related to a point $\mathbf{r}$ in space, the integration of Fukui functions (or dual descriptor) on an atomic domain Ω_k will provide the aforementioned numerical values [10, 11]. In the case of Fukui functions, the integrals that allow turning functions into the numerical values (Fukui indexes), which will be associated with the kth atom, are the following:

$$\{\int f^{+}(\mathbf{r})\, d\mathbf{r}\}_{\Omega_k} = f_k^{+};\ \ \{\int f^{-}(\mathbf{r})\, d\mathbf{r}\}_{\Omega_k} = f_k^{-} \quad \textbf{(Equation S26)}$$

where f_k^{+} and f_k^{-} correspond to the Fukui indexes on the kth atom [11]. By following the same procedure, the operational formula of the dual descript index is given by:

$$f_k^{(2)} = \left\{\int f^{(2)}(\mathbf{r})d\mathbf{r}\right\}_{\Omega_k} = \{\int f^{+}(\mathbf{r})d\mathbf{r} - \int f^{-}(\mathbf{r})d\mathbf{r}\}_{\Omega_k} = f_k^{+} - f_k^{-} \quad \textbf{(Equation S27)}$$

The unit of dual descriptor index is e^{-1}. Nevertheless, neither dual descriptor nor Fukui functions can be used to compare local reactivities among different molecules because condensed values become insignificant as the molecule's size increases. The local hyper-softness (LHS) mends this intrinsic behavior of the dual descriptor and Fukui functions [12-15]. LHS is a local reactivity descriptor that considers the molecular size and whose operational formula based on the FMOA is presented as follows:

$$s^{(2)}(\mathbf{r}) \approx S^2 f^{(2)}(\mathbf{r}) = (\varepsilon_{LUMO} - \varepsilon_{HOMO})^{-2} \cdot (|\psi_{LUMO}(\mathbf{r})|^2 - |\psi_{HOMO}(\mathbf{r})|^2) \quad \textbf{(Equation S28)}$$

Where S^2 is the squared global softness. $s^{(2)}(\mathbf{r})$ is defined in terms of LUMO and HOMO energies under the assumption that Koopmans' theorem is satisfied. Its unit is $e^3 hartree^{-2} bohr^{-3}$.

After integrating within the atomic domain Ω_k, we get the local hyper-softness index of the *k*th atom:

$$s_k^{(2)} = \left\{\int s^{(2)}(\mathbf{r})d\mathbf{r}\right\}_{\Omega_k} = (\varepsilon_{LUMO} - \varepsilon_{HOMO})^{-2} \cdot (f_k^{+} - f_k^{-}) \quad \textbf{(Equation S29)}$$

Its unit is $e^3 hartree^{-2}$. This descriptor's advantage is that it allows comparing local reactivities among different molecules; hence, we used it in the present work to assess the local reactivity of molecules under analysis [12, 15].

3D maps of local hyper-softness

The most accurate way to compute any reactivity descriptor of the Conceptual DFT implies using the finite difference approximation (FDA) because of the discrete nature of N, the number of electrons. However, a second approximation can be used when frontier molecular orbitals rule the local and global reactivity so that when this second approximation is employed for computing global reactivity descriptors, we must resort to the Koopmans' theorem. When local reactivity descriptors are computed under this second approximation, we must resort to the frontier molecular orbital approximation

(FMOA). Before resorting to the FMOA to produce a 3D picture of the local hyper-softness (LHS), comparing FDA and FMOA is a good practice.

FDA to obtain a 3D picture of LHS

$$\left(\frac{\partial^2 E}{\partial N^2}\right)^{-1}_{\upsilon(\mathbf{r})} = S = (I_1 - A_1)^{-1} \text{ (\textbf{Equation S30})}$$

Where $I_1 = E(N-1) - E(N)$ (**Equation S31**) and $I_2 = E(N-2) - E(N-1)$ (**Equation S32**) are the first and second vertical ionization potentials. While $A_1 = E(N) - E(N+1)$ (**Equation S33**) and $A_2 = E(N+1) - E(N+2)$ (**Equation S34**) are the first and second vertical electron affinities. $E(N-2)$, $E(N-1)$, $E(N)$, $E(N+1)$, and $E(N+2)$ correspond to the total energy of the system with $N-2$, $N-1$, N, $N+1$, and $N+2$ electrons, respectively computed with the molecular structure optimized with N electrons.

$$\left(\frac{\partial^2 \rho(\mathbf{r})}{\partial N^2}\right)_{\upsilon(\mathbf{r})} = f^{(2)}(\mathbf{r}) = \rho_{N+1}(\mathbf{r}) - 2\rho_N(\mathbf{r}) + \rho_{N-1}(\mathbf{r}) \text{ (\textbf{Equation S35})}$$

$$\left(\frac{\partial^3 E}{\partial N^3}\right)_{\upsilon(\mathbf{r})} = \gamma = 0.5(I_1 + A_1 - I_2 - A_2) \text{ (\textbf{Equation S36})}$$

$$\left(\frac{\partial \rho(\mathbf{r})}{\partial N}\right)_{\upsilon(\mathbf{r})} = f(\mathbf{r}) = 0.5\big(\rho_{N+1}(\mathbf{r}) - \rho_{N-1}(\mathbf{r})\big) \text{ (\textbf{Equation S37})}$$

All these operational formulae lead to the operational formula of LHS

$$\left(\frac{\partial^2 \rho(\mathbf{r})}{\partial \mu^2}\right)_{\upsilon(\mathbf{r})} = s^{(2)}(\mathbf{r}) = S^2 f^{(2)}(\mathbf{r}) - S^3 \gamma f(\mathbf{r}) \text{ (\textbf{Equation S38})}$$

where

$$\left(\frac{\partial^2 \rho(\mathbf{r})}{\partial \mu^2}\right)_{\upsilon(\mathbf{r})} = \left(\frac{\partial \left(\frac{\partial \rho(\mathbf{r})}{\partial \mu}\right)_{\upsilon(\mathbf{r})}}{\partial \mu}\right)_{\upsilon(\mathbf{r})} \text{ (\textbf{Equation S39})}$$

with

$$\left(\frac{\partial \rho(\mathbf{r})}{\partial \mu}\right)_{\upsilon(\mathbf{r})} = \left(\frac{\partial \rho(\mathbf{r})}{\partial N}\right)_{\upsilon(\mathbf{r})} \left(\frac{\partial^2 E}{\partial N^2}\right)^{-1}_{\upsilon(\mathbf{r})} = f(\mathbf{r})S \text{ (\textbf{Equation S40})}$$

and

$$\left(\frac{\partial E}{\partial N}\right)_{\upsilon(\mathbf{r})} = \mu = -0.5(I_1 + A_1) \text{ (\textbf{Equation S41})}$$

FMOA to obtain a 3D picture of LHS

This approximation is valid when there are no essential differences between the FDA and FMOA for local reactivity descriptors. Under this approximation, the $S^3\gamma f(\mathbf{r})$ the term is canceled since γ equals zero.

$$\left(\frac{\partial^2 E}{\partial N^2}\right)^{-1}_{\upsilon(\mathbf{r})} = S \approx (\varepsilon_{LUMO} - \varepsilon_{HOMO})^{-1} \text{ (Equation S42)}$$

where ε_{LUMO} and ε_{HOMO} are the energies of LUMO and HOMO, respectively.

$$\left(\frac{\partial^2 \rho(\mathbf{r})}{\partial N^2}\right)_{\upsilon(\mathbf{r})} = f^{(2)}(\mathbf{r}) \approx |\psi_{LUMO}(\mathbf{r})|^2 - |\psi_{HOMO}(\mathbf{r})|^2 \text{ (Equation S43)}$$

and $|\psi_{LUMO}(\mathbf{r})|^2$ and $|\psi_{HOMO}(\mathbf{r})|^2$ are the electron densities of LUMO and HOMO, respectively.

$$\left(\frac{\partial^3 E}{\partial N^3}\right)_{\upsilon(\mathbf{r})} = \gamma \approx 0.5(-\varepsilon_{HOMO} - \varepsilon_{LUMO} + \varepsilon_{HOMO} + \varepsilon_{LUMO}) = 0 \text{ (Equation S44)}$$

All these operational formulae lead to the operational formula of LHS

$$s^{(2)}(\mathbf{r}) \approx S^2 f^{(2)}(\mathbf{r}) = \frac{(|\psi_{LUMO}(\mathbf{r})|^2 - |\psi_{HOMO}(\mathbf{r})|^2)}{(\varepsilon_{LUMO} - \varepsilon_{HOMO})^2} \text{ (Equation S45)}$$

AOMix software

AOMix software demands the use of basis sets that do not include diffuse functions to compute condensed values of Fukui functions because diffuse functions yield mistakes in the results concerning local reactivities. Besides, AOMix resorts to the FMOA to compute those values and not FDA. Then the FMOA based on the B3LYP/6-311G(d,p) level of theory was employed to produce condensed values of LHS as quoted by **Table S9** and **Table S10**. After comparing these different pictures, we can infer that our results reveal a correct trend of local reactivity.

Section Schemes

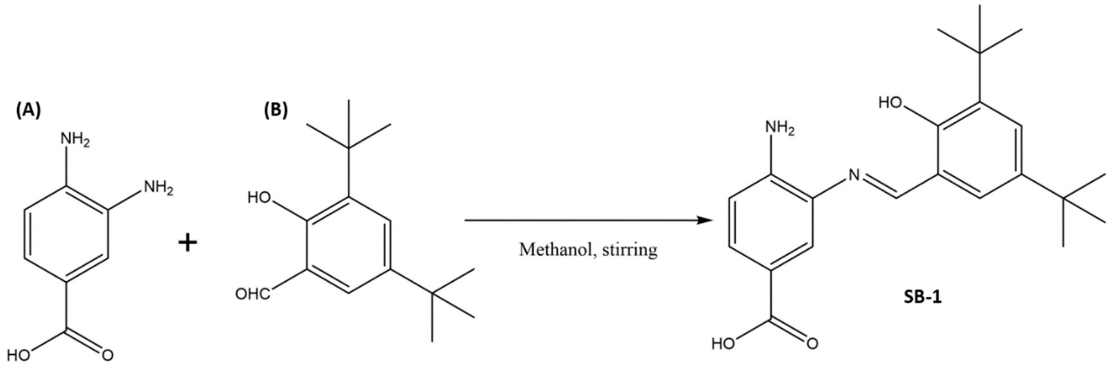

Scheme S1. Synthetic route and chemical structure of **SB-1**. The precursors A (3,4-diaminobenzoic acid) and B (3,5-di-*tert*-butyl-2-hydroxybenzaldehyde) are shown.

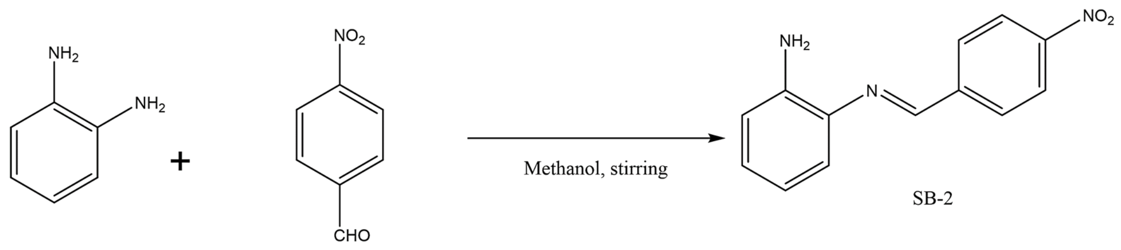

Scheme S2. Synthetic route and chemical structure of **SB-2**.

Section Figures.

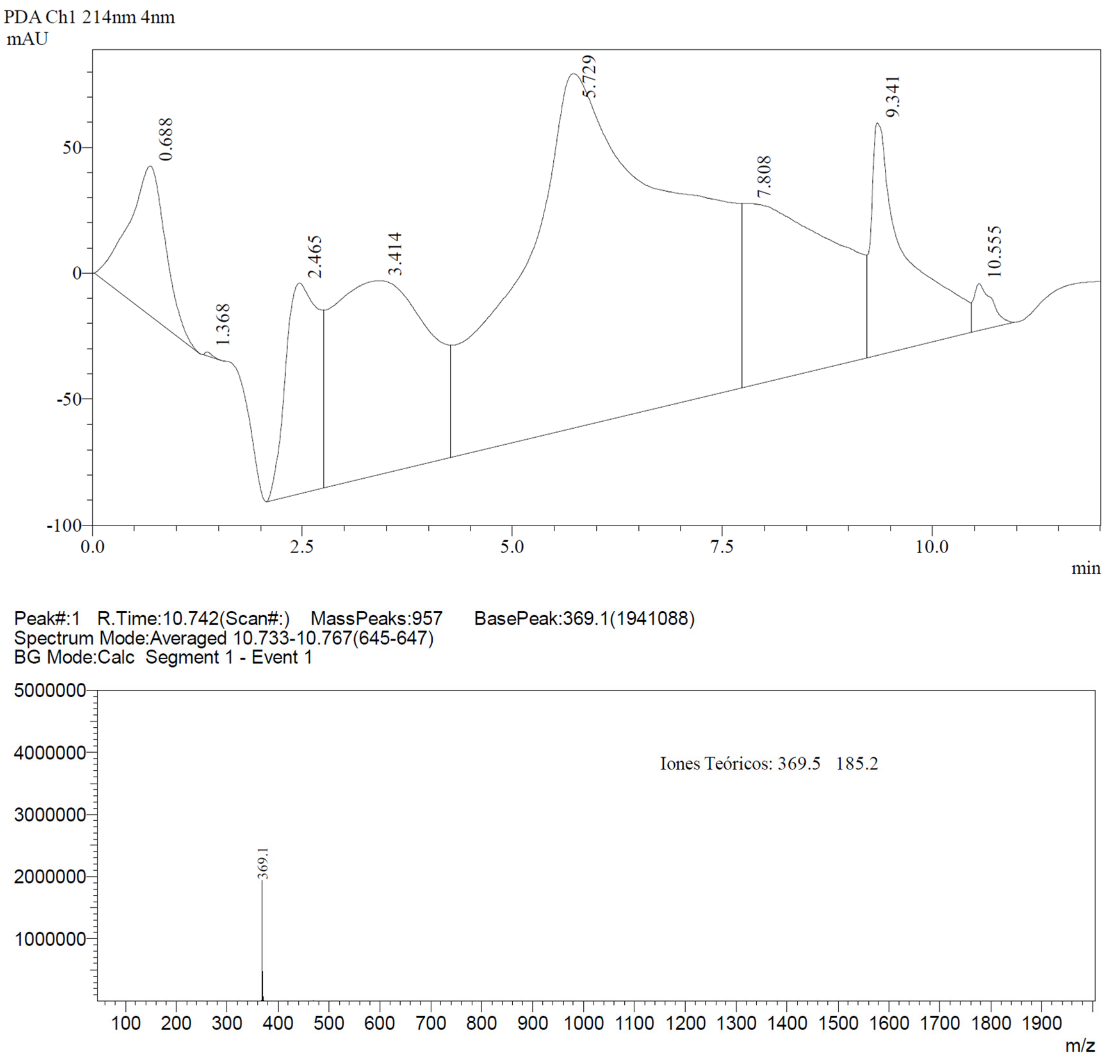

Figure S1a. Electrospray mass spectroscopy (ESMS) for **SB-1**.

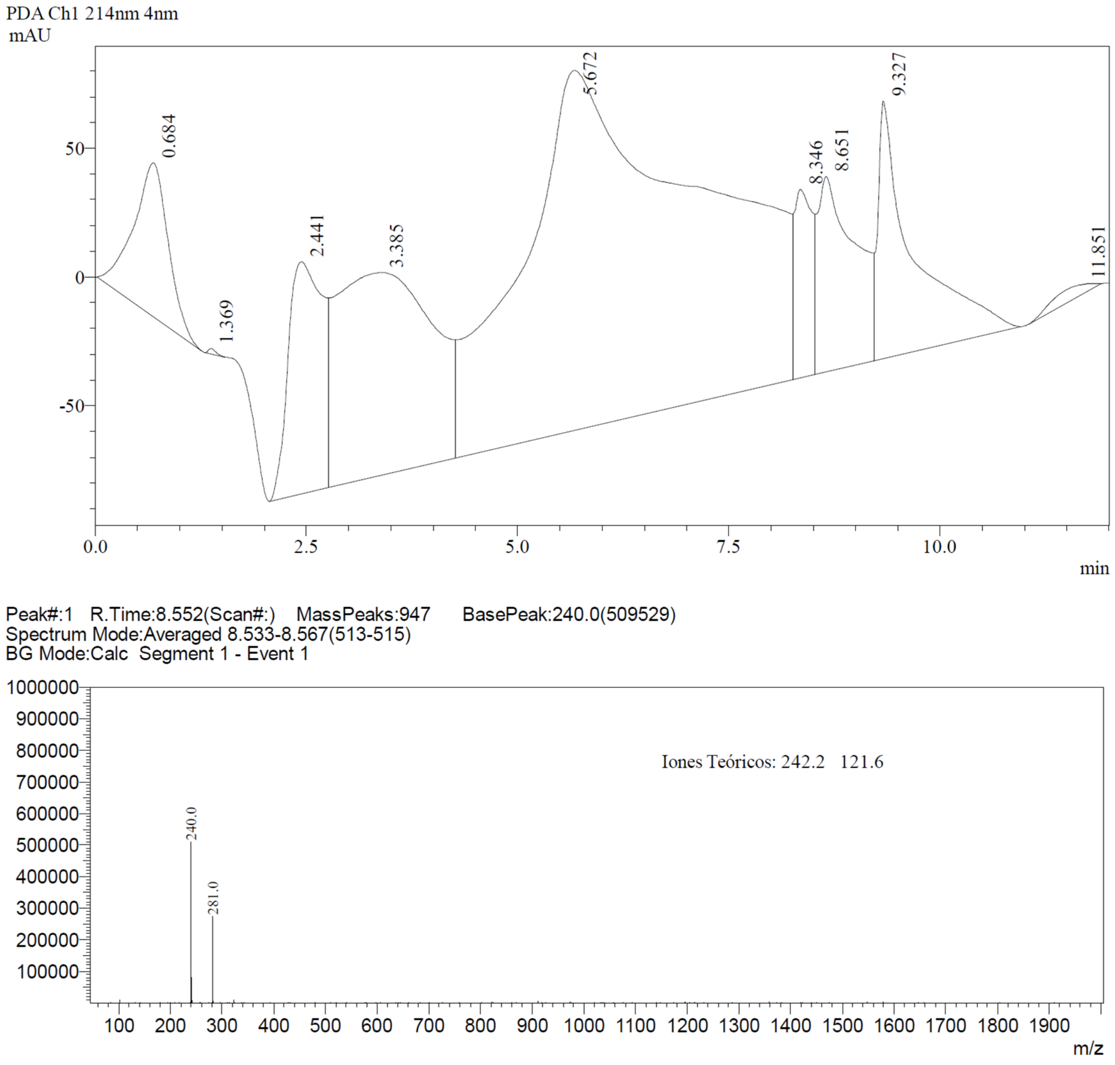

Figure S1b. Electrospray mass spectroscopy (ESMS) for **SB-2**.

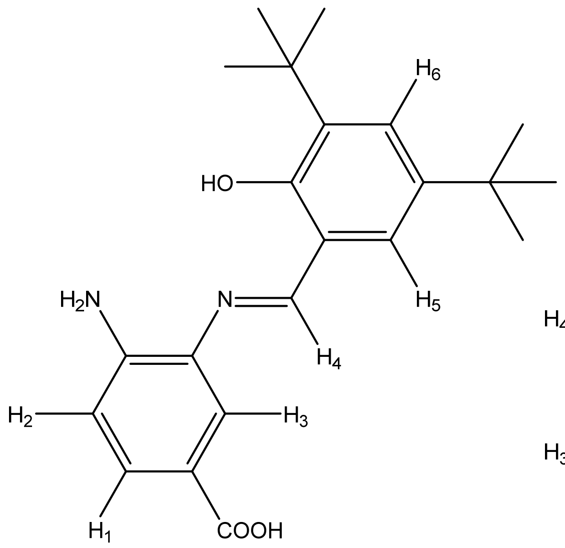

SB-1

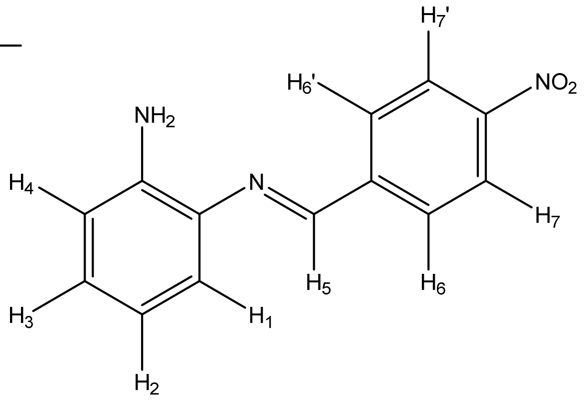

SB-2

Figure S2. Numbering of protons used in this study (arbitrary numbering).

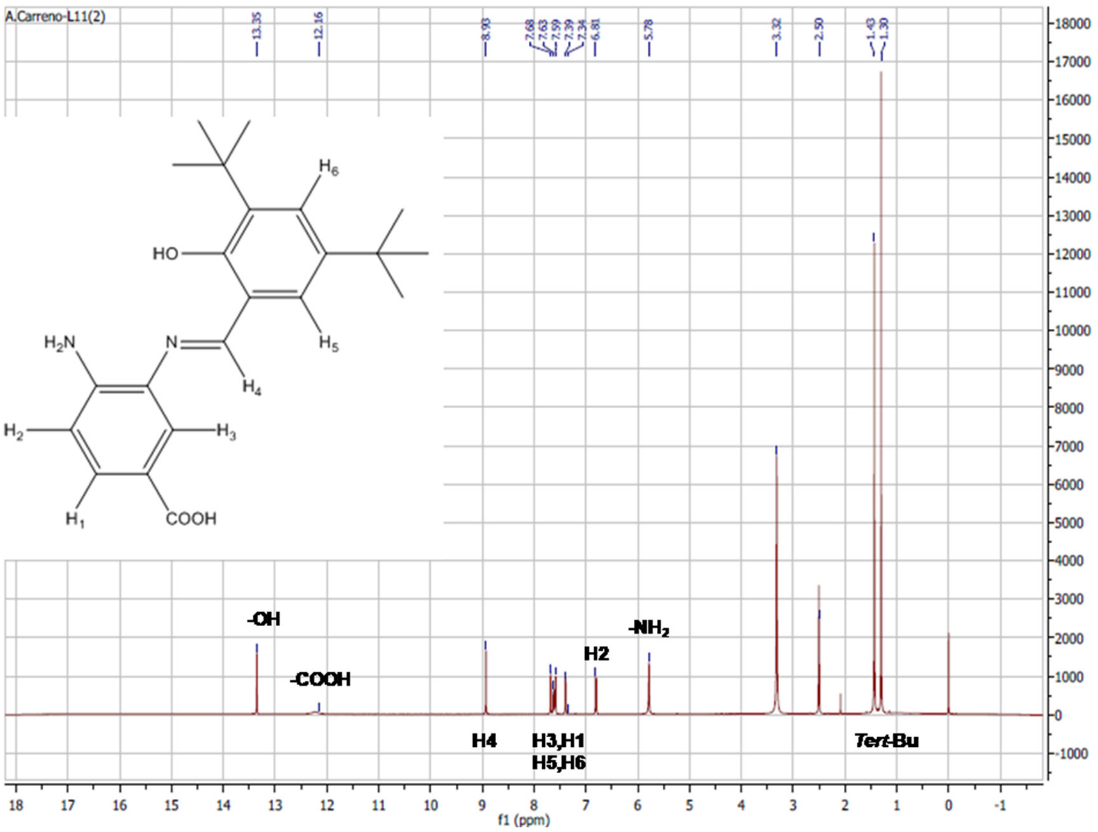

Figure S3. ^{1}H NMR of **SB-1**.

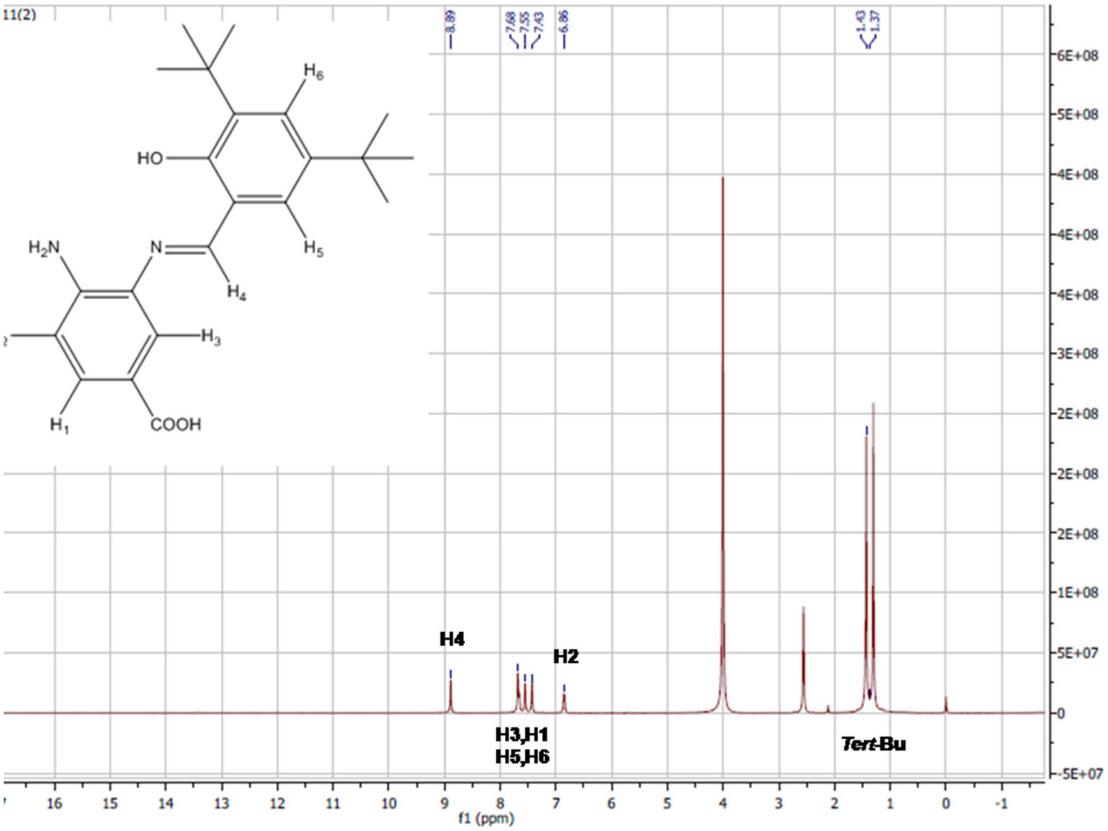

Figure S4. ^{1}H NMR with D_2O Exchange for **SB-1**.

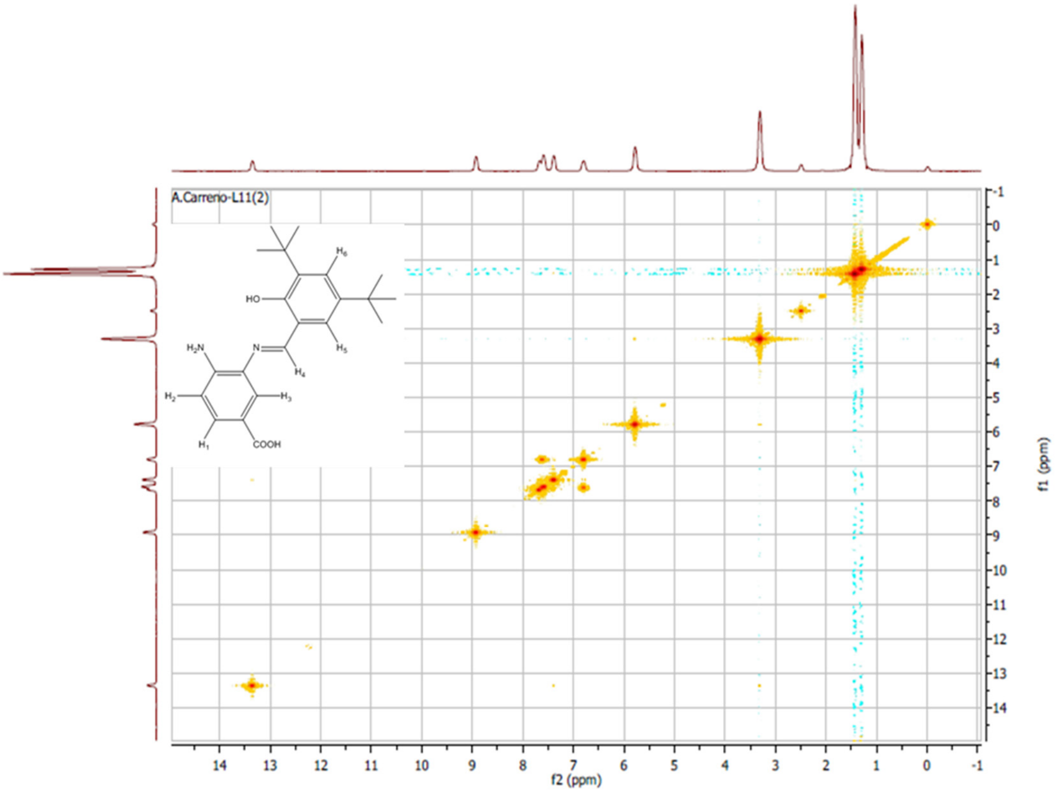

Figure S5. HHCOSY for **SB-1**.

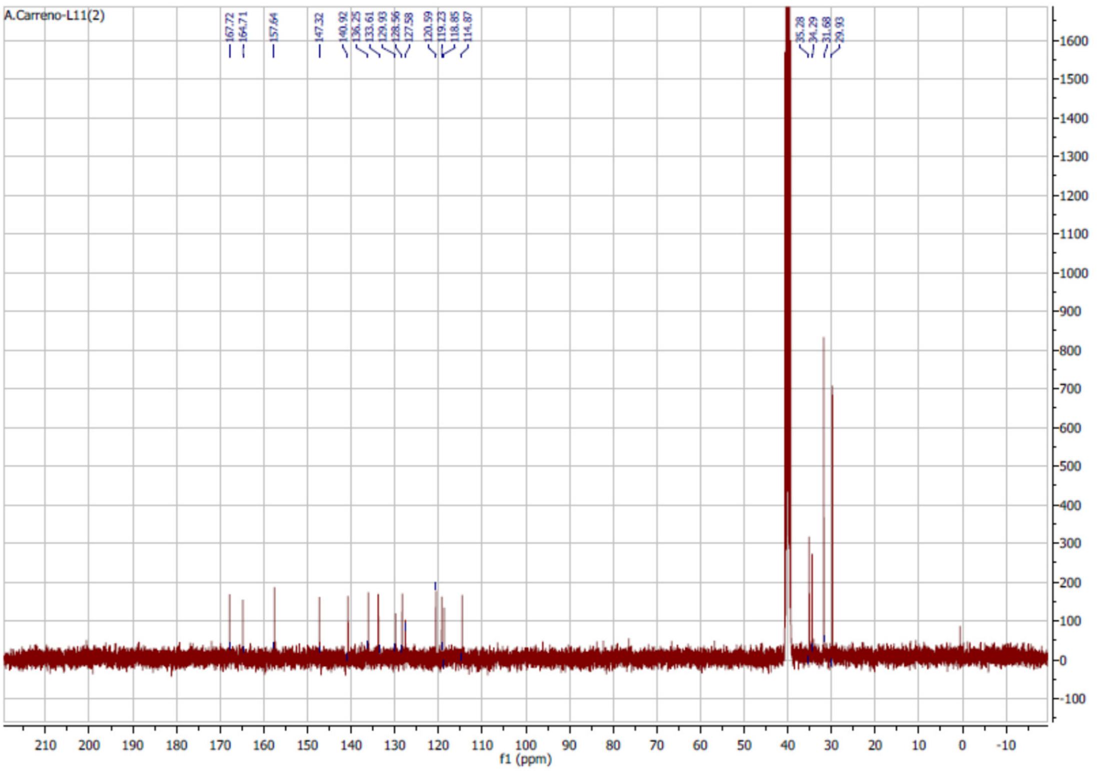

Figure S6. ^{13}C NMR for **SB-1**.

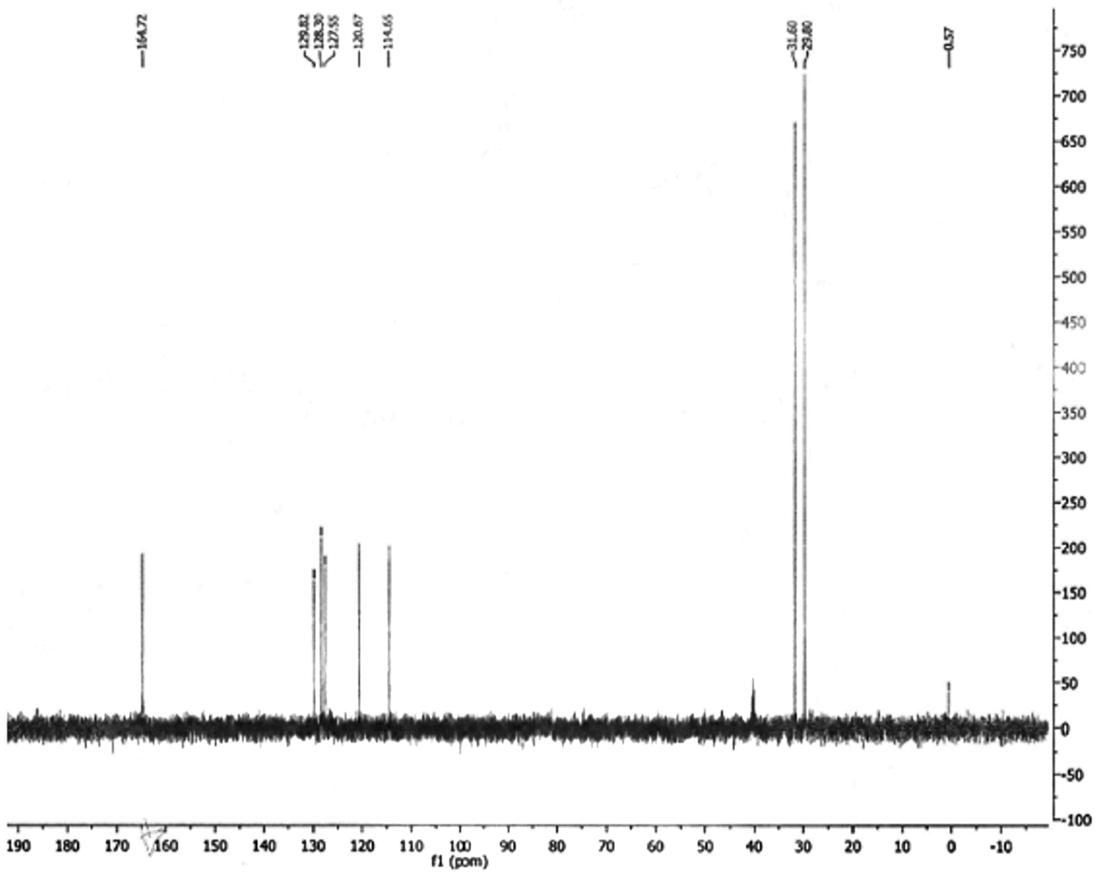

Figure S7. DEPT for **SB-1**.

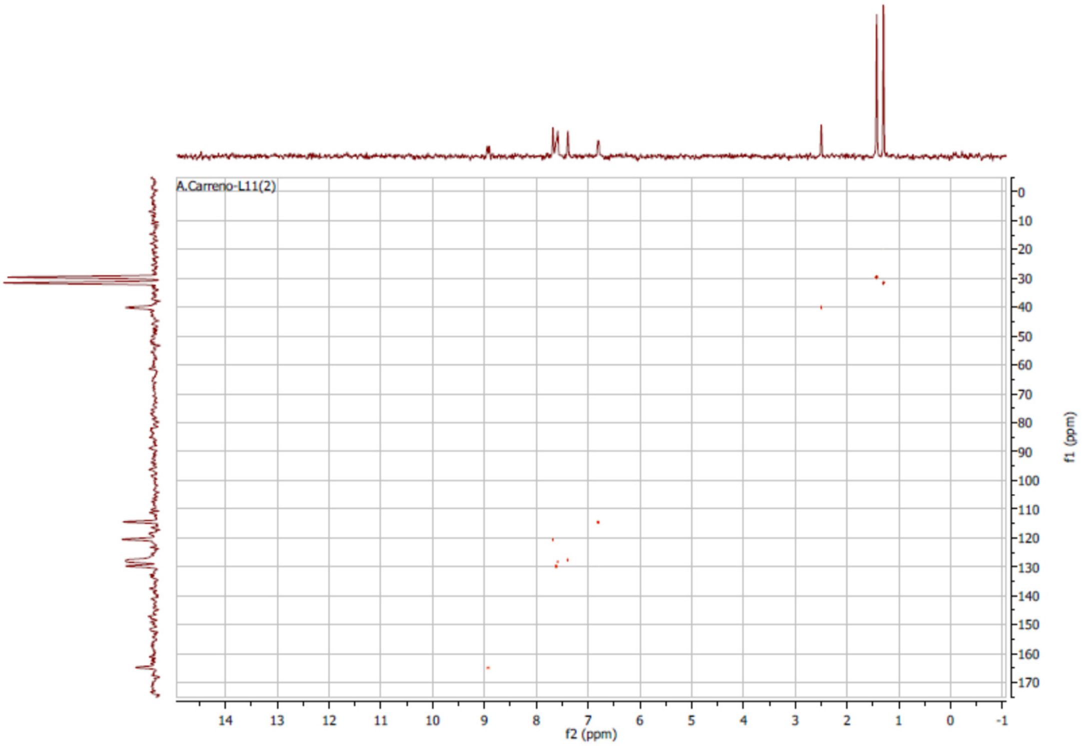

Figure S8. CHCOSY for **SB-1**.

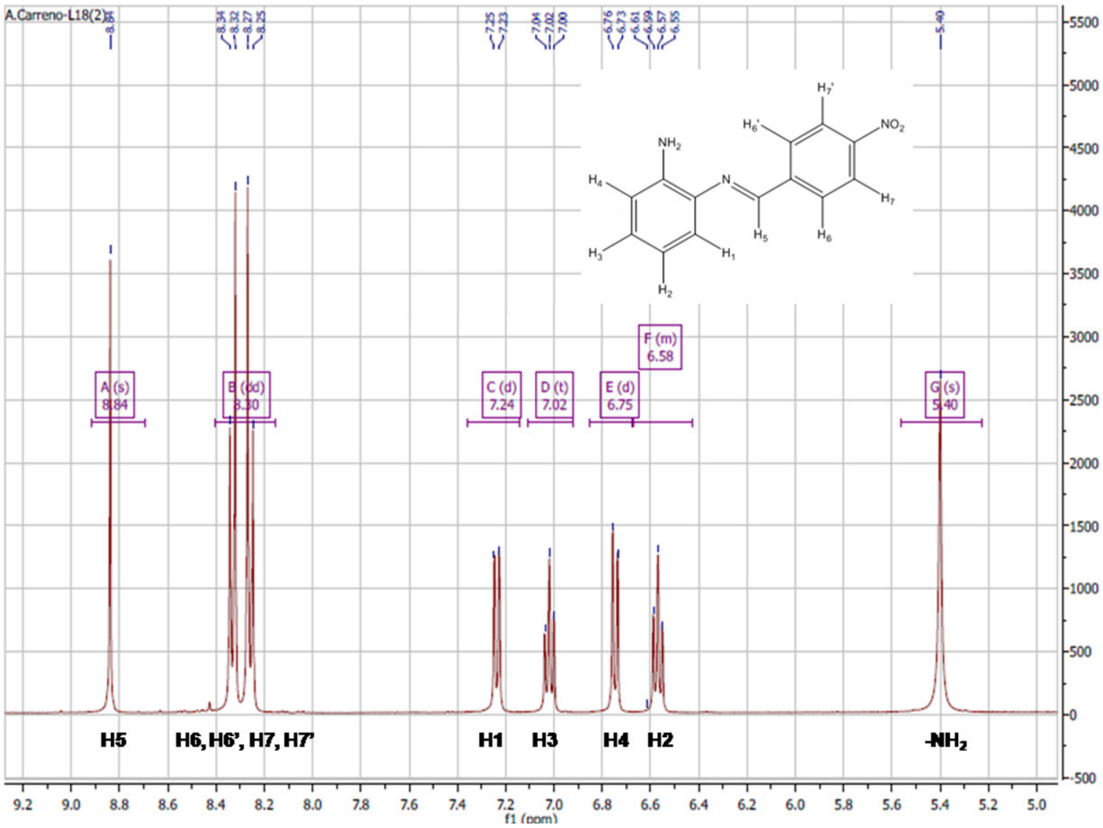

Figure S9. ¹H NMR for **SB-2** (expanded aromatic region)

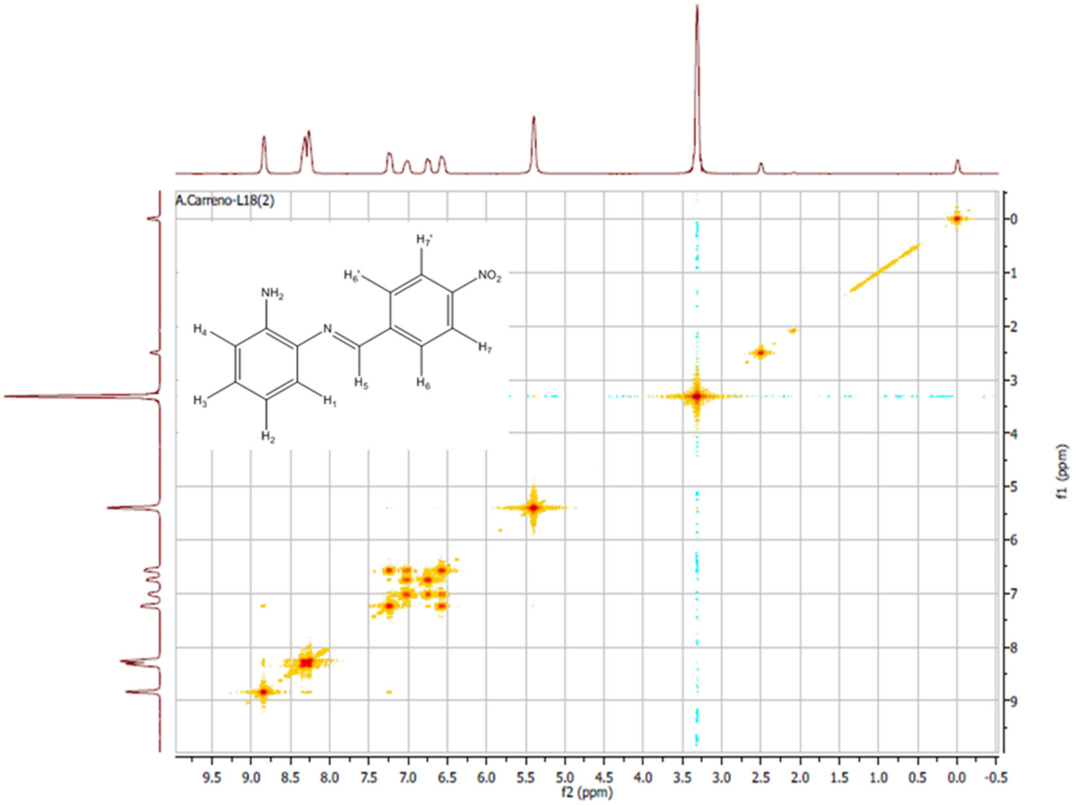

Figure S10. HHCOSY for **SB-2.**

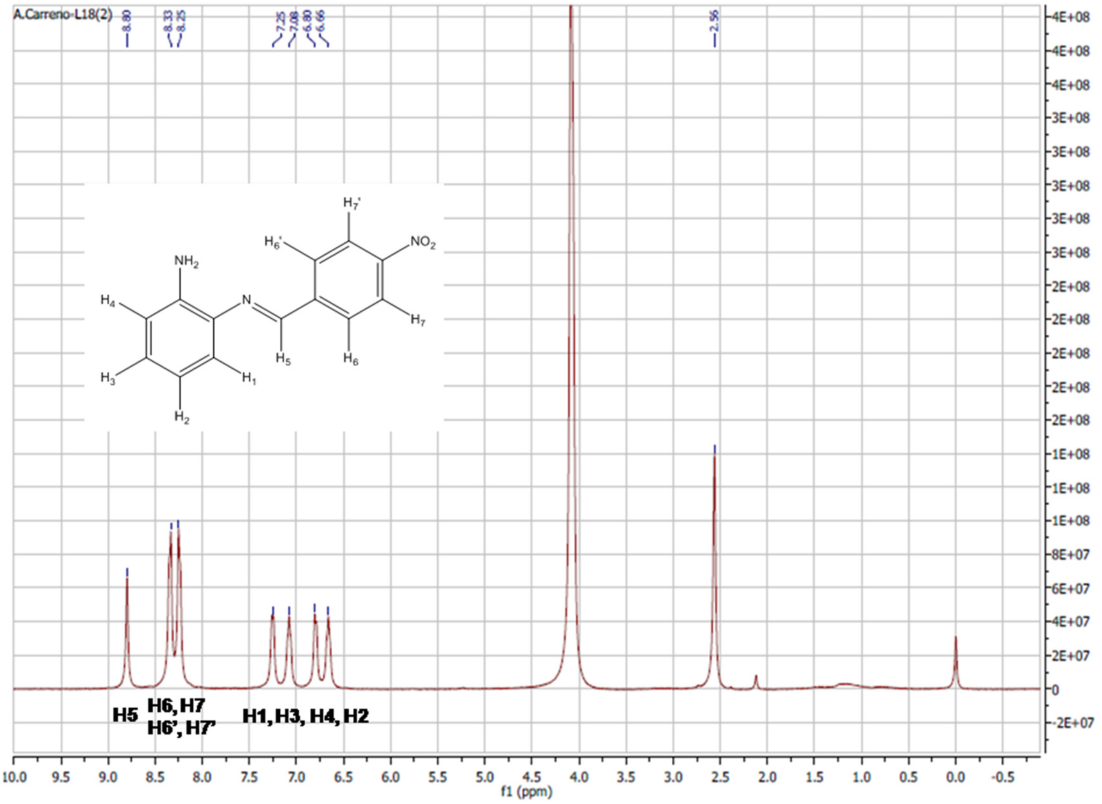

Figure S11. ^{1}H NMR with D_2O Exchange for **SB-2**.

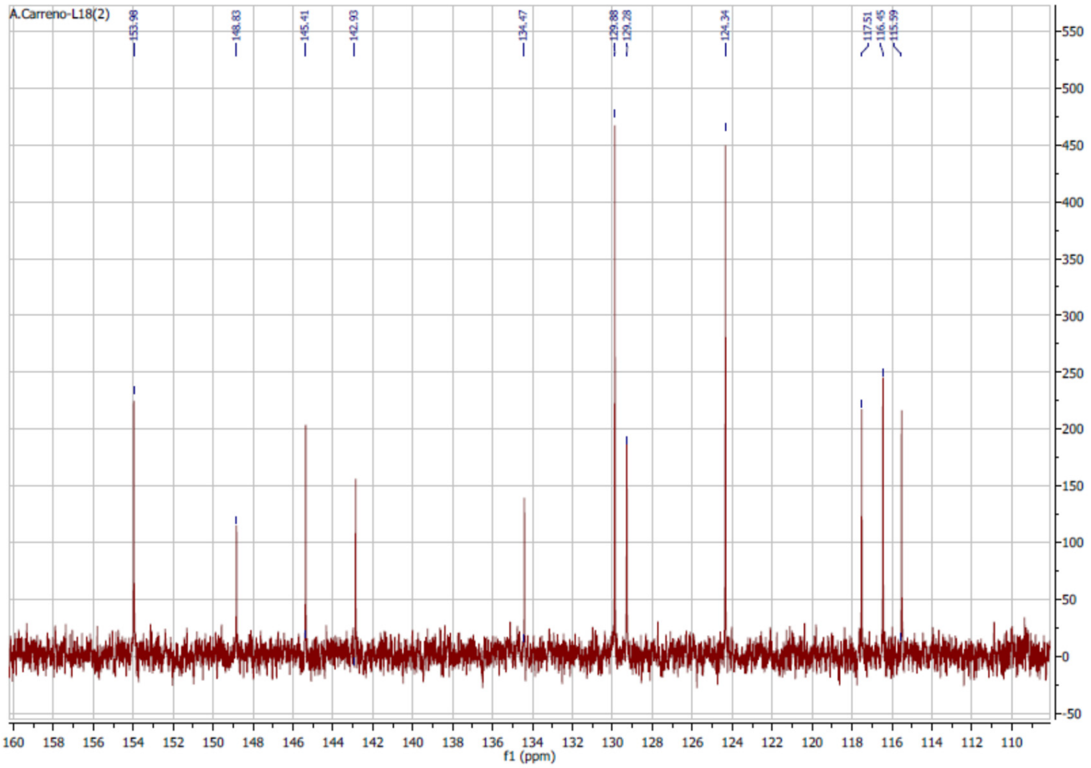

Figure S12. ^{13}C NMR for **SB-2**.

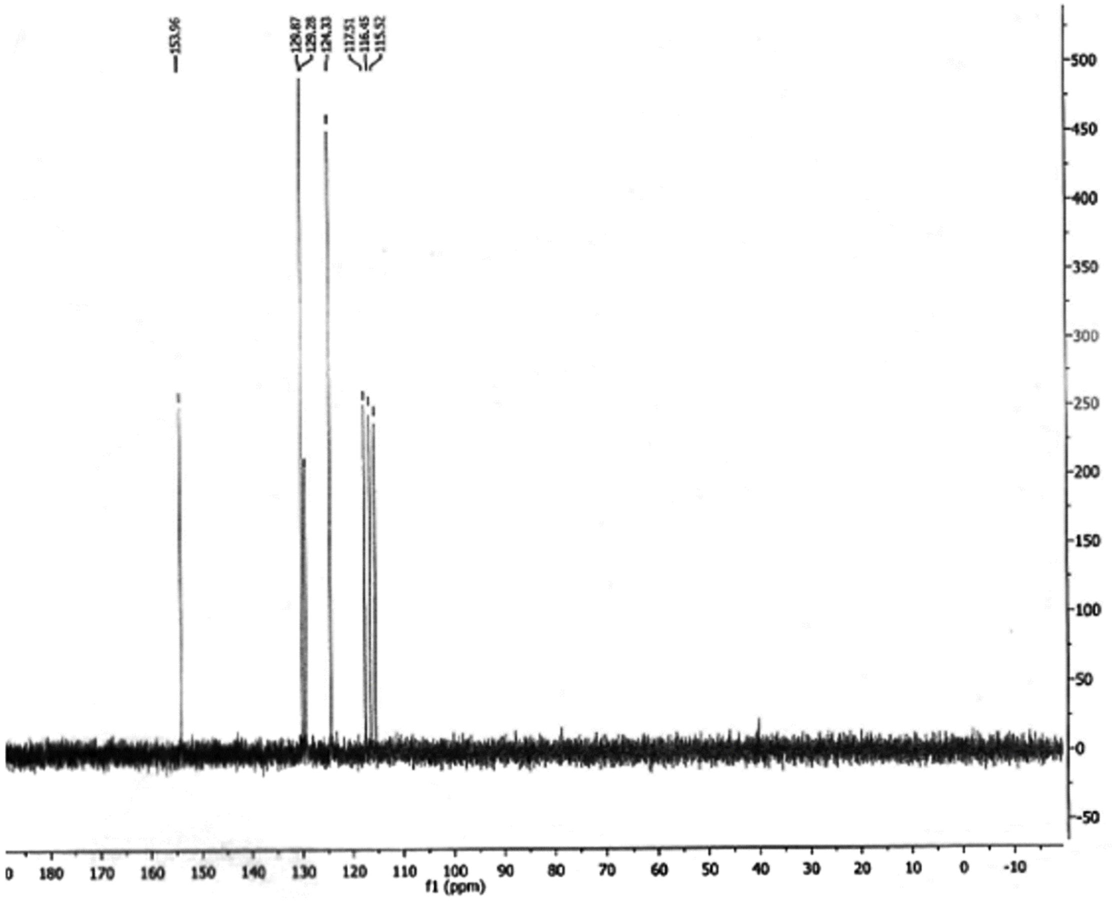

Figure S13a. DEPT for **SB-2**.

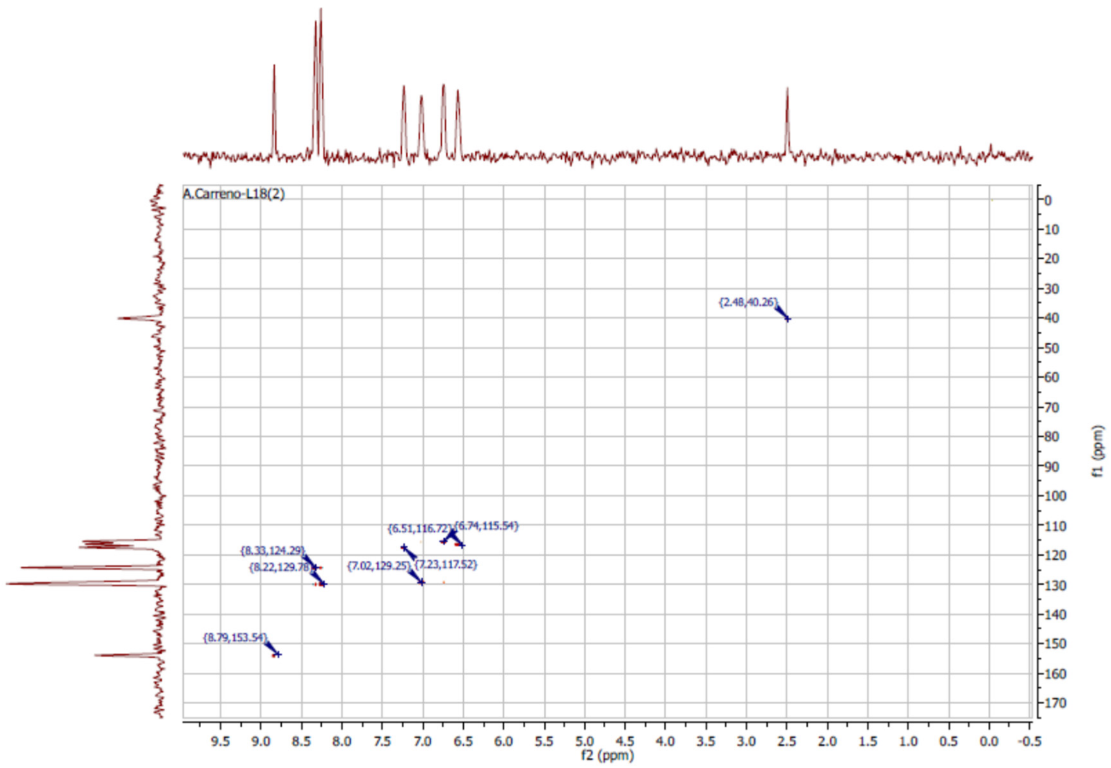

Figure S13b. CHCOSY for **SB-2**.

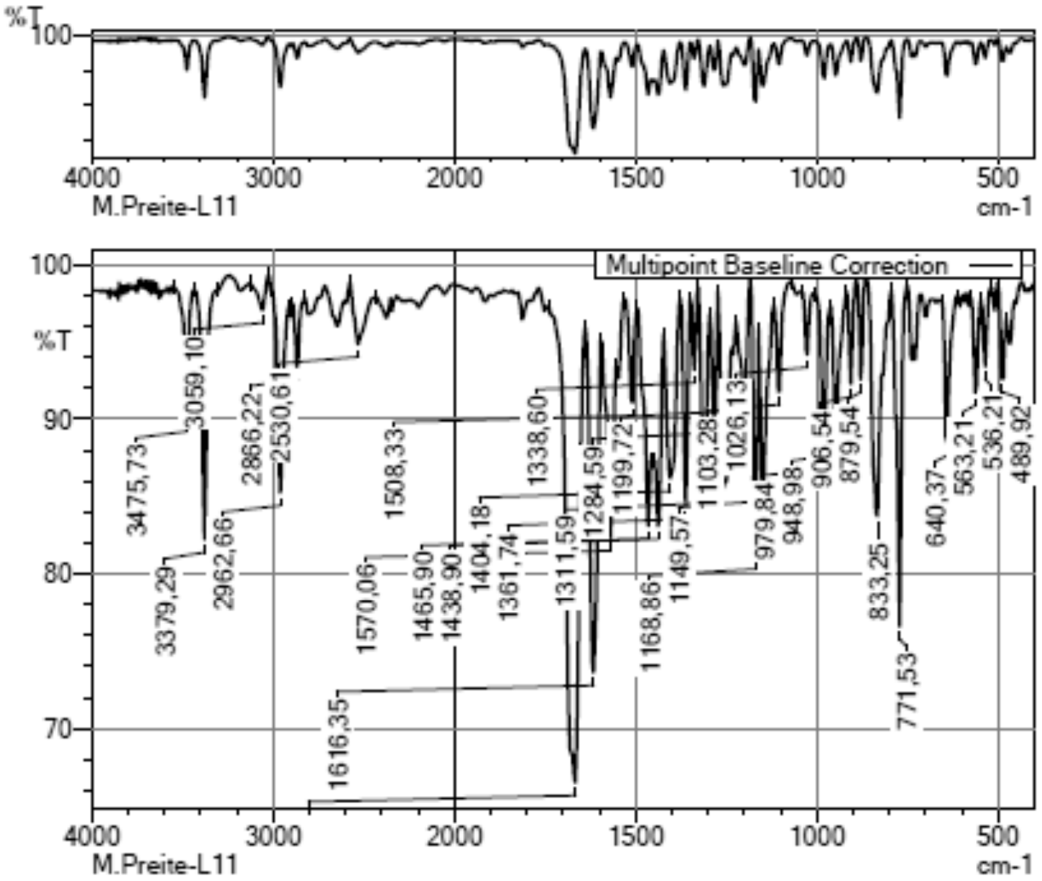

Figure S14. FTIR for **SB-1**.

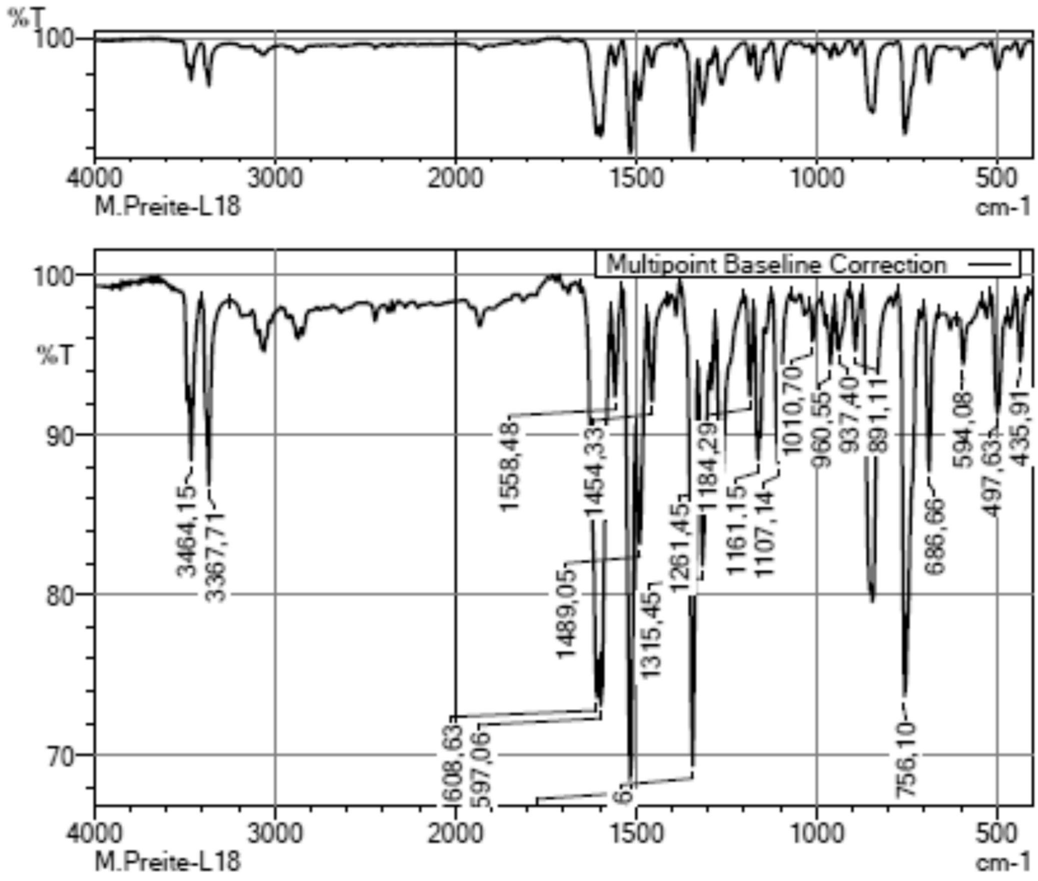

Figure S15. FTIR for **SB-2**.

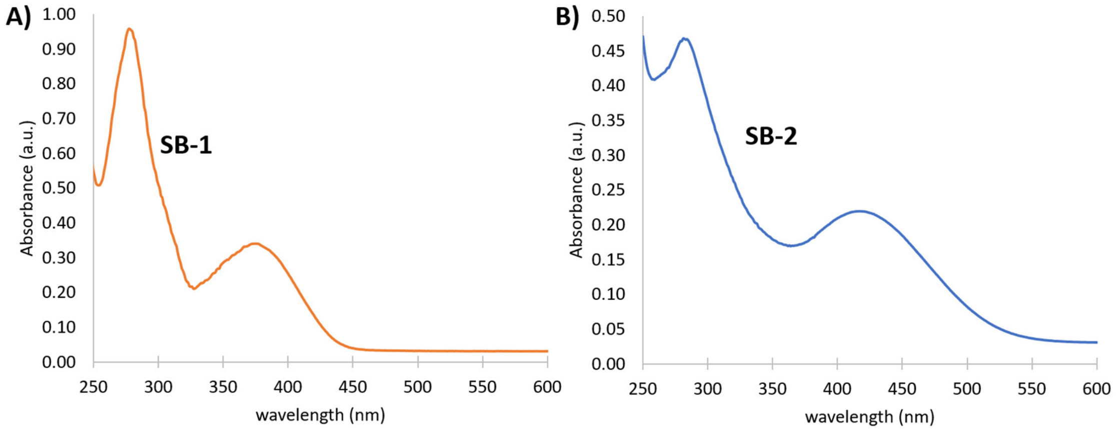

Figure S16. UV-vis spectrum for **SB-1** (A) or **SB-2** (B) in methanol at room temperature.

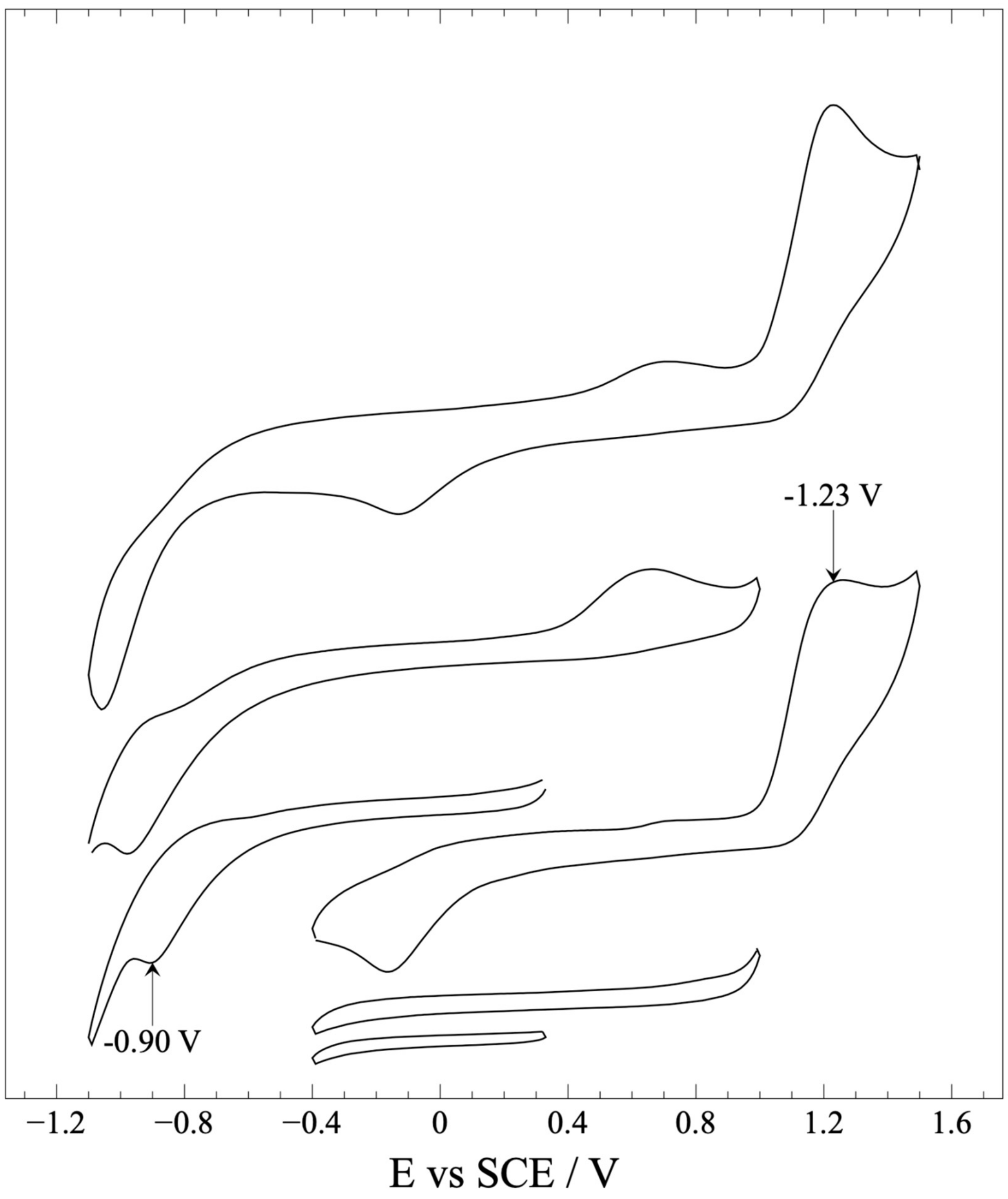

Figure S17. Working-window study for **SB-1**. Interface: Same as **Figure 2**.

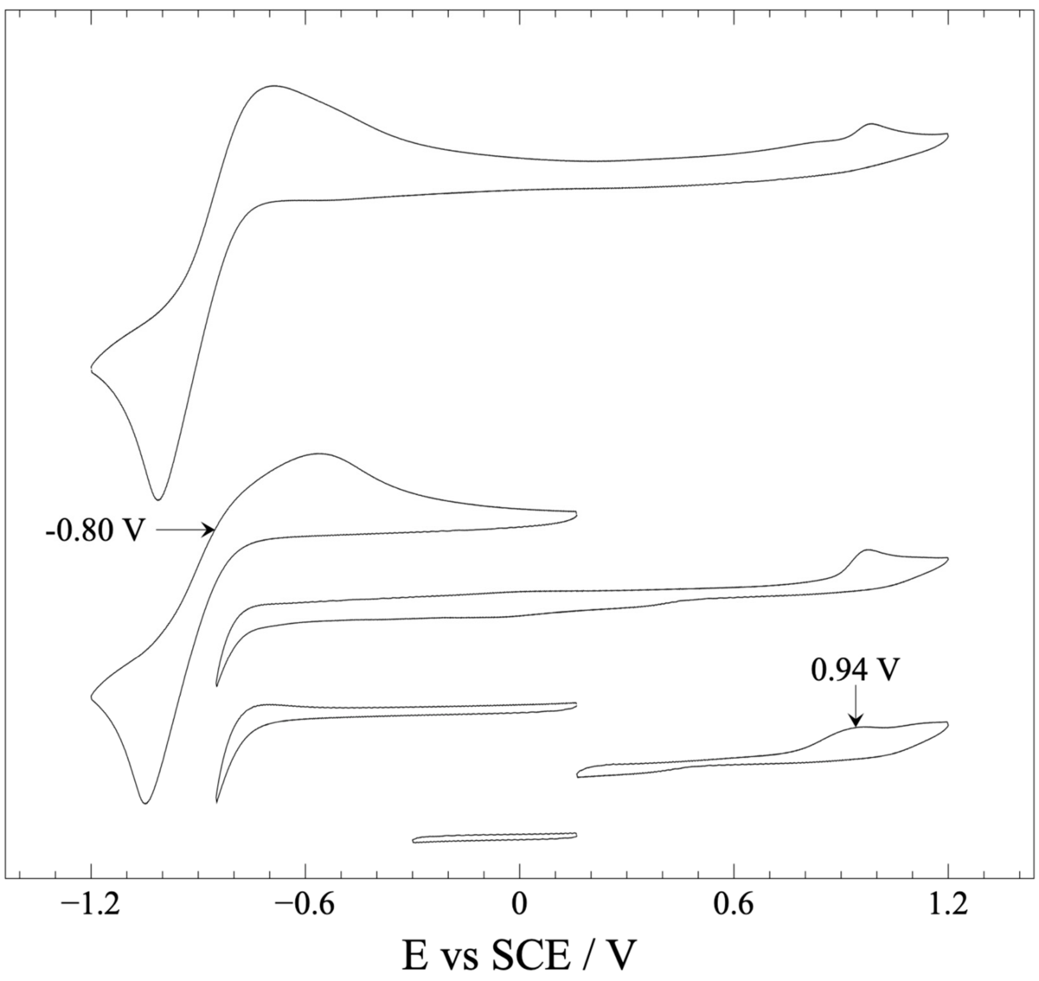

Figure S18. Working-window study for **SB-2**. Interface: Same as **Figure 2**.

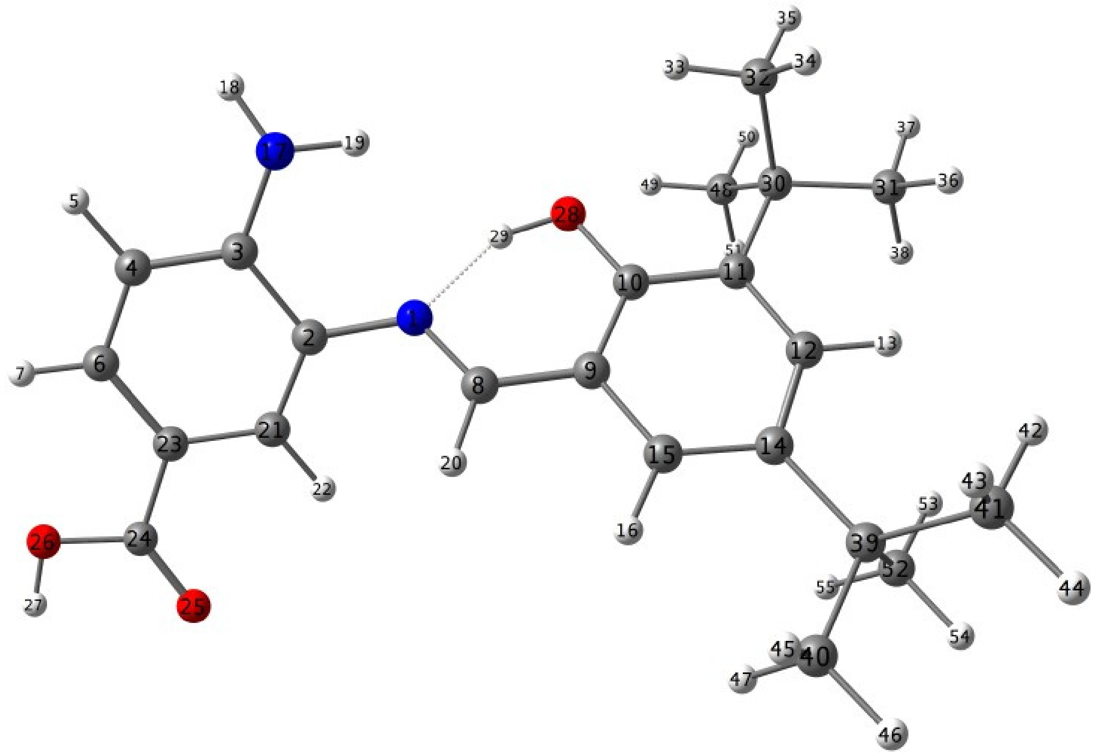

Figure S19. Geometry optimization of **SB-1**.

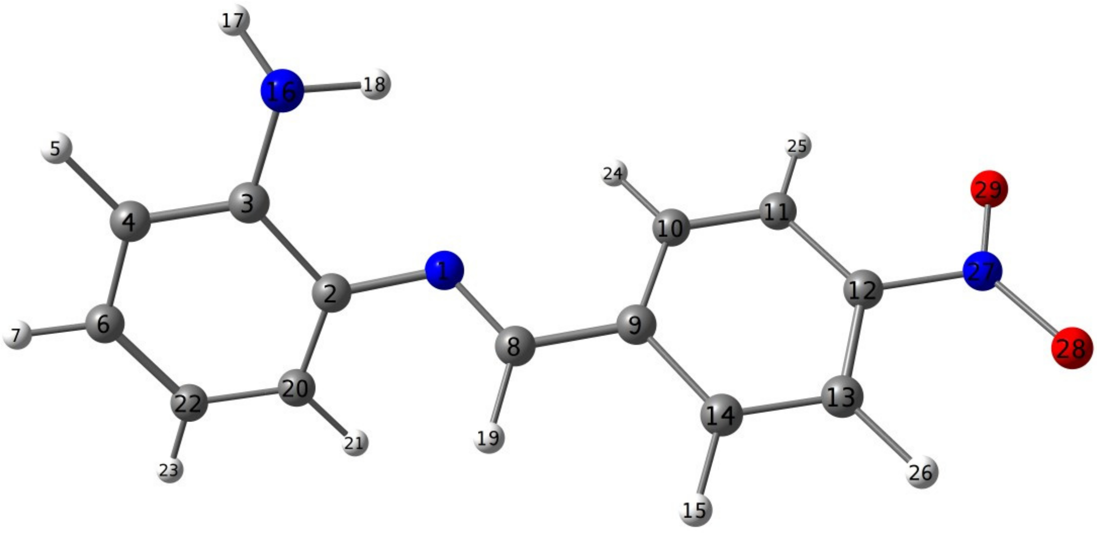

Figure S20. Geometry optimization of **SB-2**.

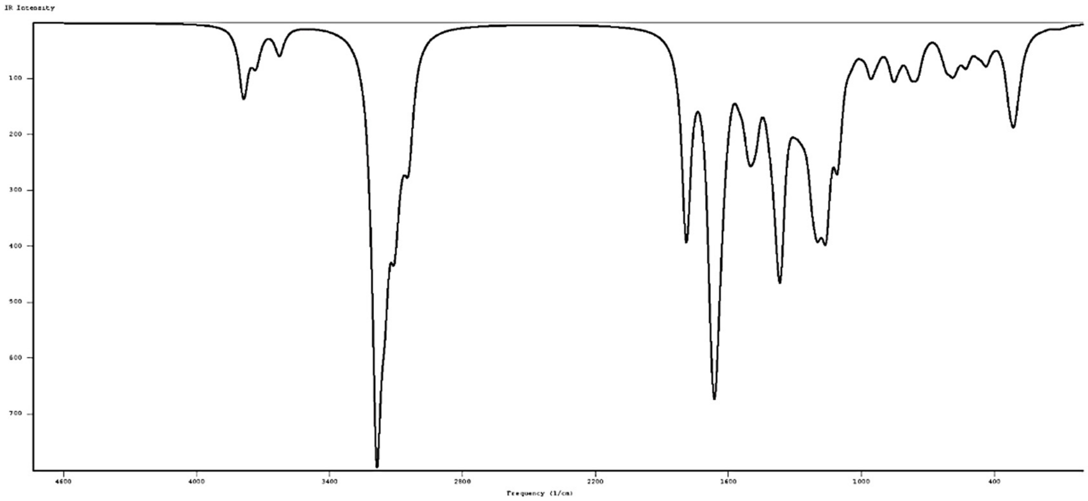

Figure S21. Theoretical infrared spectra for **SB-1**.

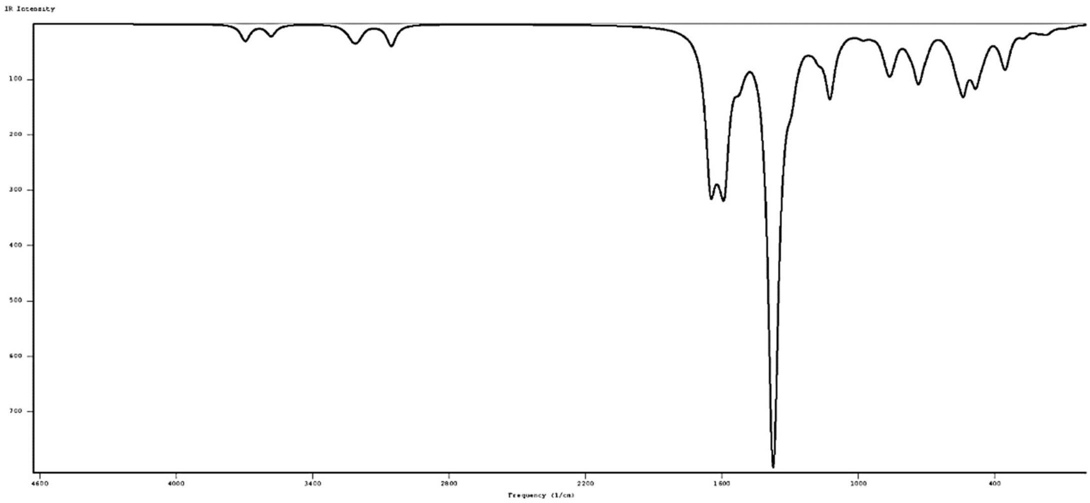

Figure S22. Theoretical infrared spectra for **SB-2**.

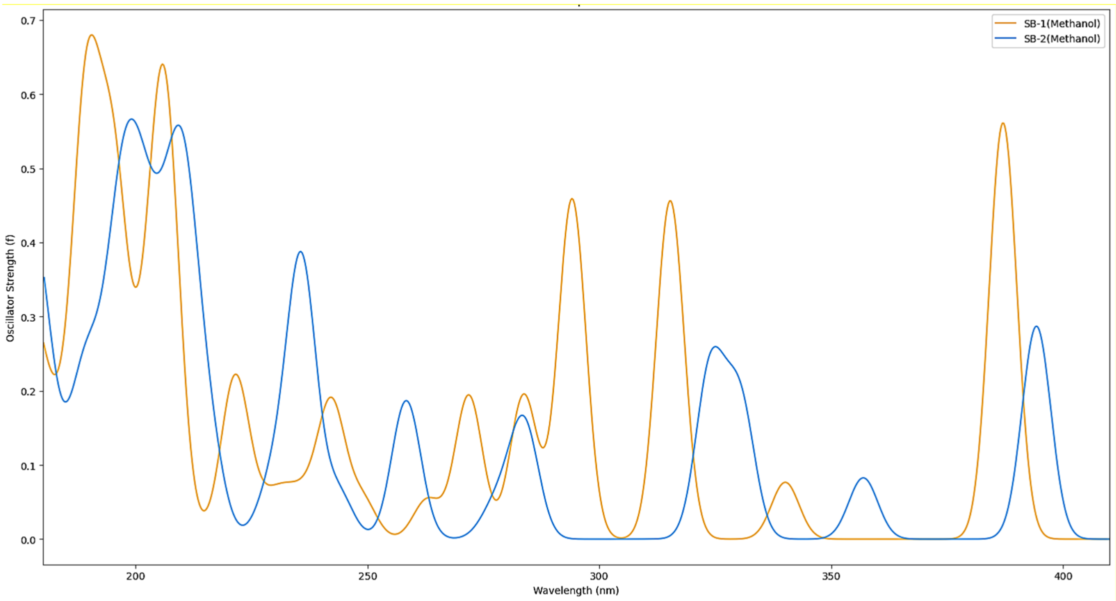

Figure S23. Computed UV-vis in methanol for **SB-1** (orange) and **SB-2** (blue).

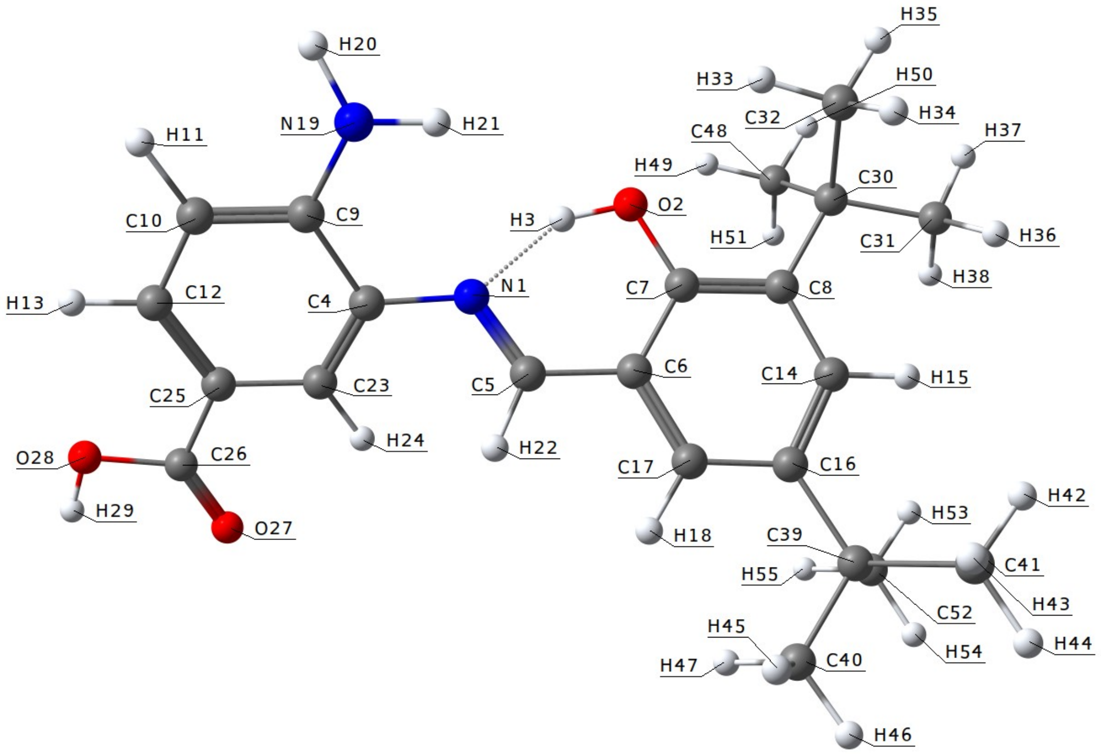

Figure S24. Numbering of atoms for NBO analysis for **SB-1**.

FDA	FMOA	
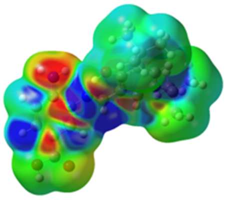	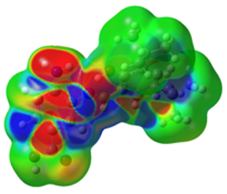	**B3LYP/6-311++G(d,p)**
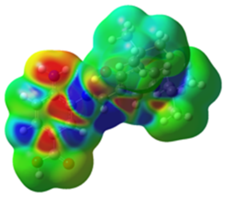	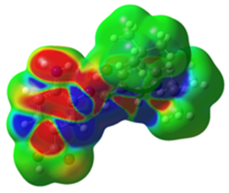	**B3LYP/6-311G(d,p)**

Figure S25. Maps of LHS for **SB-1** ranging from -0.001 $e^3 \cdot hartree^{-2} \cdot bohr^{-3}$ to 0.001 $e^3 \cdot hartree^{-2} \cdot bohr^{-3}$ and projected onto an isodensity of 0.001 $e \cdot bohr^{-3}$.

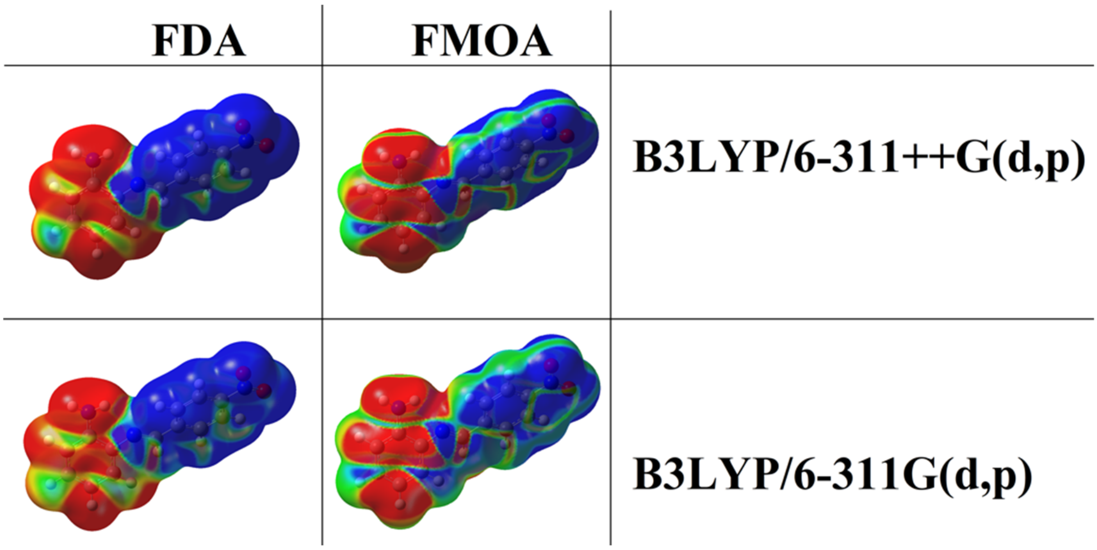

Figure S26. Maps of LHS for **SB-2** ranging from -0.001 $e^3 \cdot hartree^{-2} \cdot bohr^{-3}$ to 0.001 $e^3 \cdot hartree^{-2} \cdot bohr^{-3}$ and projected onto an isodensity of 0.001 $e \cdot bohr^{-3}$

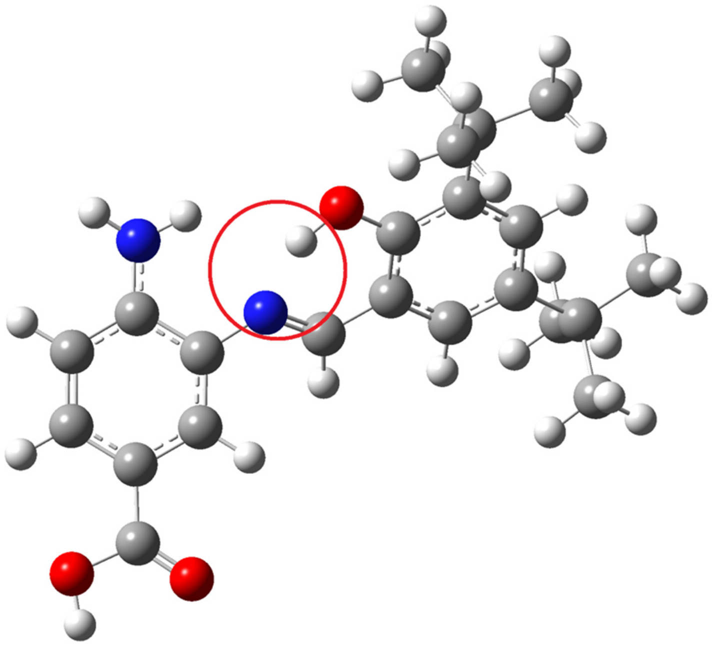

Figure S27. Nitrogen (blue-colored sphere) and hydrogen (white-colored sphere) atoms of **SB-1** inside the red-colored circle. Bond order analyses of Wiberg and Mayer indicate there is a hydrogen bond between these two atoms.

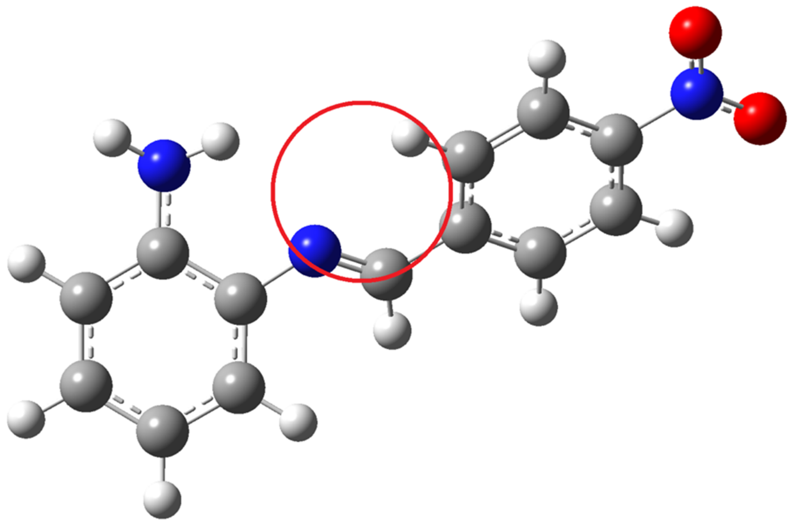

Figure S28. Nitrogen (blue-colored sphere) and hydrogen (white-colored sphere) atoms of **SB-2** inside the red-colored circle. Bond order analyses of Wiberg and Mayer indicate there is no hydrogen bond between these two atoms.

Section Tables

Table S1. Optimized geometry parameters for **SB-1** in the ground state**.**

**Bond length (Å)**	**C3-N17**	**C8=N1**	**C10-O28**	**N1···H29**	**O28-H29**
	1.368	1.286	1.347	1.712	0.992
**Bond angle (°)**	**H18-N17-H19**	**C2-N1=C8**	**C10-O28-H29**	**O28-H29···N1**	**C9-C8=N1**
	116.40	121.75	106.95	150.43	123.80

Table S2. Optimized geometry parameters for **SB-2** in the ground state**.**

**Bond length (Å)**	**C3-N16**	**C8=N1**	**C12-N27**	**N27-O29**	**N27-O28**
	1.379	1.273	1.472	1.224	1.224
**Bond angle (º)**	**H17-N16-H18**	**C2-N1=C8**	**C9-C8=N1**	**O28-N27-O29**	
	114.85	121.88	122.93	124.61	

Table S3. Calculated frequencies (cm^{-1}) for **SB-1** and **SB-2** (X: absence of the functional group in the Schiff base).

FTIR spectrum	SB-1	SB-2
**OH (COOH)**	3787	X
**NH_2 (as)**	3732	3696
**NH_2(s)**	3626	3582
**OH (IHB)**	3187	X
**C-H(C8=N1)**	3075	3054
**C=O (COOH)**	1790	X
**C8=N1**	1680	1696
**NO_2**	X	1378

Table S4. Calculated absorption wavelength λ (nm), oscillator strengths (f), and corresponding transition and assignment for **SB-1** and **SB-2** (methanol as solvent).

Schiff base	λ	f	Major contribution	Assignment
**SB-1**	387	0.5614	H → L (97.83 %)	π → π*
	294	0.4588	H-2 → L (88.74 %)	
**SB-2**	394	0.2871	H-1 → L (96.96 %)	
	236	0.3352	H → L+4 (89.69 %)	

Table S5. Donor-acceptor interaction analysis from NBO calculations for **SB-1**.

Donor	Acceptor	ΔE (kcal/mol)
N1 (LP)	O2-H3 (BD*)	26.9
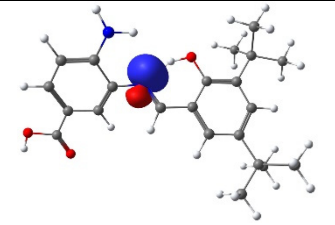	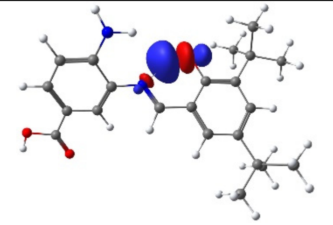	
O2 (LP)	C6-C7 (BD*)	39.4
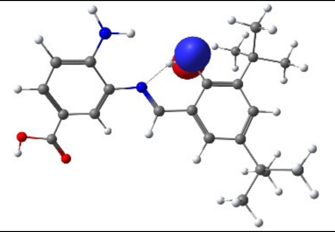	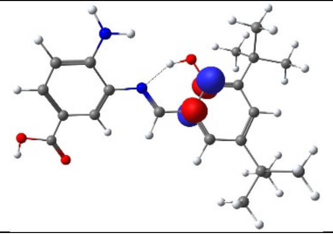	
N19 (LP)	C9-C10 (BD*)	41.1
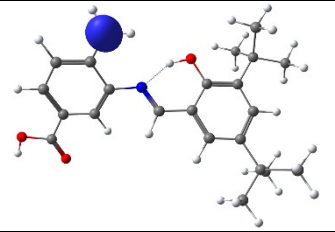	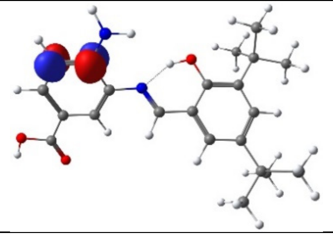	
O27 (LP)	C26-O28	35.8
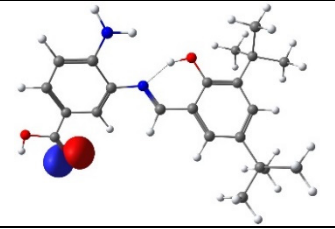	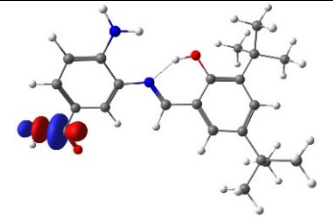	
O28 (LP)	C26-O27	44.9
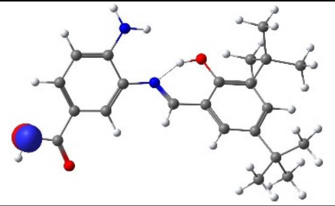	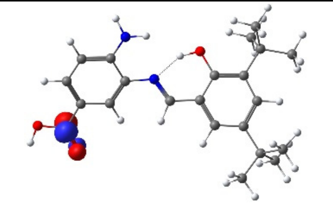	
N1-C5 (BD)	C4-C23 (BD*)	11.1
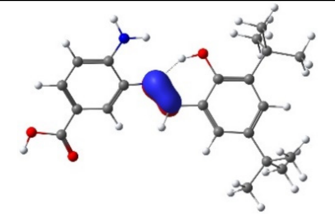	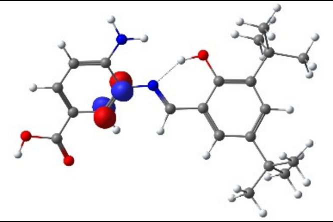	
C4-C23 (BD)	N1-C5 (BD*)	10.9

Donor	Acceptor	ΔE (kcal/mol)
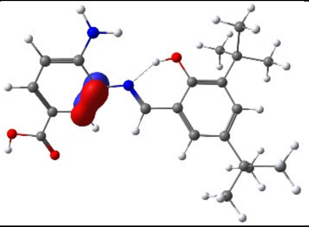	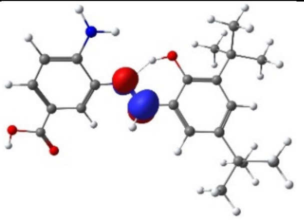	
C6-C7 (BD)	N1-C5 (BD*)	25.2
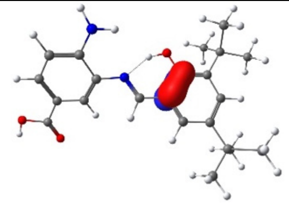	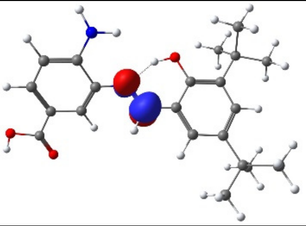	

Table S6. Condensed values of LHS of **SB-1** in $e^3 \cdot hartree^{-2}$.

Atom	Value
C1	-6.08
C in COOH	-0.29
O in CO from COOH	-1.14
O in OH from COOH	-0.27
H in COOH	0.00
C2	2.46
C3	-4.52
C4	-3.20
C5	-1.51
C6	0.71
N in NH_2	-8.48
H in NH_2	-0.12
N in N=C	5.69
C in N=C	12.69
C1’	-1.40
C2’	0.77
O in OH-C2’	-1.56
C3’	0.23
C4’	4.48
C5’	-2.52
C6’	4.31

Table S7. Condensed values of LHS of **SB-2** in $e^3 \cdot hartree^{-2}$.

Atom	Value
C1	-9.47
C2	-14.19
C3	1.63
C4	-16.88
C5	-1.85
C6	-7.12
N in NH_2	-24.72
H in NH_2	-0.41
H in NH_2	-0.53
N in N=C	6.55
C in N=C	0.65
C1’	9.30
C2’	1.27
C3’	6.70
C4’	6.10
C5’	4.65
C6’	2.19
N in NO_2	12.76
O in NO_2	11.69
O in NO_2	11.91

Notice that both hydrogen atoms in NH_2 present similar values, so it is not worth distinguishing each of them.

Table S8. Wiberg and Mayer's bond order analyses between nitrogen and hydrogen atoms are highlighted in figures **S21** and **S22.**

	Wiberg	Mayer
**SB-1**	0.157	0.157
	**Wiberg**	**Mayer**
**SB-2**	0.034	0.034

Table S9. Resistance/Sensitivity profile of strains used in this study (Gram-positive bacteria)

	Bacitracin (10 U)		Vancomycin (30 U)		Penicillin (10 U)		Chloramphenicol (30 U)		Tetracycline (30 U)	
**Strain**	**Halo (mm)**	**Resistance**	**Halo (mm)**	**Resistance**	**Halo (mm)**	**Resistance**	**Halo (mm)**	**Resistance**	**Halo (mm)**	**Resistance**
Staphylococcus aureus	7	R	21	S	7	R	19	S	30	S
Enterococcus faecalis	7	R	23	S	17	S	28	S	13	R

R: Resistant; S: Sensitive; I: Intermediate resistance; halo diameter was measured

Biochemical tests:

Strain	Gram	Catalase	Bacitracin	Coagulase	Hemolysis	Bile esculin
Staphylococcus aureus	Positive	Positive	Resistant	Positive	Gamma	NT
*Enterococcus faecalis**	Positive	Negative	Resistant	NT	Gamma	Positive

NT: Non tested

*: Identified by MALDI-TOF

Table S10. Resistance/Sensitivity profile of strains used in this study (Gram-negative bacteria)

	Bacitracin (10 U)		Ciprofloxacin (5 U)		Ampicillin (10 U)		Chloramphenicol (30 U)		Tetracycline (30 U)	
**Strain**	**Halo (mm)**	**Resistance**	**Halo (mm)**	**Resistance**	**Halo (mm)**	**Resistance**	**Halo (mm)**	**Resistance**	**Halo (mm)**	**Resistance**
Klebsiella pneumoniae	7	R	22	S	7	R	23	S	20	S
Escherichia coli	7	R	29	S	16	I	23	S	22	S
Salmonella Typhi	7	R	33	S	27	S	29	S	28	S
Salmonella Typhimurium	7	R	28	S	21	S	28	S	21	S

R: Resistant; S: Sensitive; I: Intermediate resistance; halo diameter was measured

Biochemical tests:

Strain	Gram	ONPG	GLU	ARA	LDC	ODC	CIT	H_2S	URE	TDA	IND	OX	NO_2
Klebsiella pneumoniae	Negative	+	+	+	+	-	+	-	+	-	-	-	+
Escherichia coli	Negative	+	+	+	-	+	-	-	-	-	+	-	+
Salmonella Typhi*	Negative	-	+	-	+	-	-	-	-	-	-	-	-
Salmonella Typhimurium**	Negative	-	+	NT	NT	NT	NT	NT	NT	NT	NT	NT	NT

+: Positive

-: Negative

NT: non tested

*: This strain corresponds to *Salmonella enterica* serovar Typhi STH2370 [16-18]

**: This strain corresponds to *Salmonella enterica* serovar Typhimurium ATCC14028s [19, 20]

ONPG: (β-galactosidase; Ortho-Nitrophenyl-βD-Galactopyrano-sidase)
GLU: (Fermentation / oxidation of glucose)
ARA: (Fermentation / oxidation of arabinose)
LCD: (Lysine Decarbox-ylase)
ODC: (Ornithine Decarboxylase)
CIT: (Citrate utilization)
H_2S: (H2S production)
URE: (urease)
TDA: (Tryptophane deaminase)
IND: (Indole production)
OX: (Cytochrome-oxidase)
NO_2: (NO_2 production)

Table S11. Serial dilutions used for MIC determination.

Well	Solute (SB-1 or SB-2) (μM)	Solvent DMSO (% v/v) in LB broth
*a**	500	37.5**
b	250	18.75**
c	125	9.375**
d	62.5	4.6875**
e	31.25	2.34375
f	15.625	1.171875
g	7.8125	0.5859375
h	3.90625	0.29296875

* In the well *a*, there are 500 μM of **SB-1** or **SB-2**, prepared in a solution of 37.5% (v/v) DMSO in Luria-Bertani (LB) broth

** These concentrations of DMSO kill bacteria. Thus, we considered that the compound under study exerted an antimicrobial effect only when solutions from wells *e*, *f*, *g*, or *h* inhibit bacteria growth.

References

1. Morell, C.; Grand, A.; Toro-Labbe, A.; Chermette, H., Is hyper-hardness more chemically relevant than expected? *J Mol Model* **2013,** 19, (7), 2893-900.
2. Parr, R. G.; Szentpály, L. v.; Liu, S., Electrophilicity Index. *Journal of the American Chemical Society* **1999,** 121, (9), 1922-1924.
3. Gazquez, J. L.; Cedillo, A.; Vela, A., Electrodonating and electroaccepting powers. *The journal of physical chemistry. A* **2007,** 111, (10), 1966-70.
4. Chattaraj, P. K.; Chakraborty, A.; Giri, S., Net electrophilicity. *The journal of physical chemistry. A* **2009,** 113, (37), 10068-74.
5. Chermette, H., Chemical reactivity indexes in density functional theory. *Journal of Computational Chemistry* **1999,** 20, (1), 129-154.
6. Geerlings, P.; De Proft, F.; Langenaeker, W., Conceptual density functional theory. *Chemical reviews* **2003,** 103, (5), 1793-873.
7. Sandoval-Yañez, C.; Martínez-Araya, J. I., Assessment of a set of twelve density functionals to estimate the global reactivity of myricetin through the Koopmans' theorem. *Chemical Physics Letters* **2019,** 715, 354-359.
8. Morell, C.; Grand, A.; Toro-Labbe, A., New dual descriptor for chemical reactivity. *The journal of physical chemistry. A* **2005,** 109, (1), 205-12.
9. Morell, C.; Grand, A.; Toro-Labbé, A., Theoretical support for using the Δf(r) descriptor. *Chemical Physics Letters* **2006,** 425, (4-6), 342-346.
10. Bulat, F. A.; Chamorro, E.; Fuentealba, P.; Toro-Labbé, A., Condensation of Frontier Molecular Orbital Fukui Functions. *The Journal of Physical Chemistry A* **2003,** 108, (2), 342-349.
11. Contreras, R. R.; Fuentealba, P.; Galván, M.; Pérez, P., A direct evaluation of regional Fukui functions in molecules. *Chemical Physics Letters* **1999,** 304, (5-6), 405-413.
12. Martínez-Araya, J. I., Explaining Some Anomalies in Catalytic Activity Values in Some Zirconocene Methyl Cations: Local Hyper-Softness. *The Journal of Physical Chemistry C* **2013,** 117, (47), 24773-24786.
13. Ayers, P. W.; Morell, C.; De Proft, F.; Geerlings, P., Understanding the Woodward-Hoffmann rules by using changes in electron density. *Chemistry* **2007,** 13, (29), 8240-7.
14. Morell, C.; Hocquet, A.; Grand, A.; Jamart-Grégoire, B., A conceptual DFT study of hydrazino peptides: Assessment of the nucleophilicity of the nitrogen atoms by means of the dual descriptor Δf(r). *Journal of Molecular Structure: THEOCHEM* **2008,** 849, (1-3), 46-51.
15. Cardenas, C.; Rabi, N.; Ayers, P. W.; Morell, C.; Jaramillo, P.; Fuentealba, P., Chemical reactivity descriptors for ambiphilic reagents: dual descriptor, local hypersoftness, and electrostatic potential. *The journal of physical chemistry. A* **2009,** 113, (30), 8660-7.
16. Valenzuela, C.; Ugalde, J. A.; Mora, G. C.; Alvarez, S.; Contreras, I.; Santiviago, C. A., Draft Genome Sequence of *Salmonella enterica* Serovar Typhi Strain STH2370. *Genome announcements* **2014,** 2, (1).
17. Nevermann, J.; Silva, A.; Otero, C.; Oyarzun, D. P.; Barrera, B.; Gil, F.; Calderon, I. L.; Fuentes, J. A., Identification of Genes Involved in Biogenesis of Outer Membrane Vesicles (OMVs) in Salmonella enterica Serovar Typhi. *Front Microbiol* **2019,** 10, 104.
18. Marchant, P.; Carreño, A.; Vivanco, E.; Silva, A.; Nevermann, J.; Otero, C.; Araya, E.; Gil, F.; Calderon, I. L.; Fuentes, J. A., "One for all": Functional transfer of OMV-mediated polymyxin B resistance from *Salmonella enterica* sv. Typhi Δ*tolR* and Δ*degS* to susceptible bacteria. *Front Microbiol* **2021**.
19. Jarvik, T.; Smillie, C.; Groisman, E. A.; Ochman, H., Short-term signatures of evolutionary change in the *Salmonella enterica* serovar typhimurium 14028 genome. *Journal of bacteriology* **2010,** 192, (2), 560-7.

20. Berrocal, L.; Fuentes, J. A.; Trombert, A. N.; Jofre, M. R.; Villagra, N. A.; Valenzuela, L. M.; Mora, G. C., *stg* fimbrial operon from *S.* Typhi STH2370 contributes to association and cell disruption of epithelial and macrophage-like cells. *Biological research* **2015,** 48, 34.
